# Supplementary material for: Cooperative insulation of regulatory domains by CTCF-dependent physical insulation and promoter competition
Source: Nat Commun. 2024 Aug 23;15:7258. doi: 10.1038/s41467-024-51602-4 (PMC11344162; doi:10.1038/s41467-024-51602-4)
Supplement: Supplementary file 1 — Supplementary Information [file 41467_2024_51602_MOESM1_ESM.pdf]

Supplementary Information

Synergistic insulation of regulatory domains by developmental genes and clusters of CTCF sites

Thais Ealo*[,1], Victor Sanchez-Gaya*[,#,1], Patricia Respuela[1], María Muñoz-San Martín[1,2], Elva Martin-Batista[3], Endika Haro[#,1], Alvaro Rada-Iglesias[#,1]

1. Institute of Biomedicine and Biotechnology of Cantabria (IBBTEC), CSIC/Universidad de Cantabria, Albert Einstein 22, 39011 Santander, Spain
2. Royal College of Surgeons in Ireland (RCSI), Molecular & Cellular Therapeutics, 123 St Stephens Green, Dublin 2, Ireland.
3. Centro de Biología Molecular Severo Ochoa (CBMSO), CSIC-UAM, 28029 Madrid, Spain.

* Equal contribution

# corresponding authors: endika.haro@unican.es; victor.sanchezgaya@unican.es; alvaro.rada@unican.es

Supplementary Figure 1. *Hi-C profiles at representative examples of mouse developmental TADs.*

Supplementary Figure 2. *Hi-C profiles at representative examples of human developmental TADs.*

Supplementary Figure 3. *Enriched GO categories for human TADs with different gene densities.*

Supplementary Figure 4. *Boundary-proximal housekeeping genes are located at shorter distances from TAD boundaries than boundary-proximal developmental genes.*

Supplementary Figure 5. *Insulation scores and boundary strength of TAD boundaries associated with bin 1 genes.*

Supplementary Figure 6. *RNA Pol2 pausing index (PI) for different gene categories depending on their transcriptional status and proximity to TAD boundaries.*

Supplementary Figure 7. *Developmental genes and clusters of CTCF sites are sequentially organized near human TAD boundaries.*

Supplementary Figure 8. *Orientation of CTCF sites around boundary-proximal genes.*

Supplementary Figure 9. *Conservation of CTFC clusters around the GBX2 and SIX3/SIX2 loci across vertebrates.*

Supplementary Figure 10. *Genotyping of the Δ3XCTCF, 71Kb INV and Δ3XCTCF:71Kb INV re-arrangements generated at the Gbx2/Asb18 locus.*

Supplementary Fig. 11. *Assessing the role of the CTCF site located next to the Gbx2 SE in the control of Gbx2 expression.*

Supplementary Fig. 12. *The orientation of Gbx2 does not have a major role in the insulation of its own regulatory domain.*

Supplementary Figure 13. *Capture-C experiments in Δ3XCTCF, 71Kb INV and Δ3XCTCF:71Kb INV mESC.*

Supplementary Figure 14. *Genotyping of the ΔPromGbx2 and Δ3XCTCF:ΔPromGbx2 re-arrangements generated at the Gbx2/Asb18 locus.*

Supplementary Figure 15. *Capture-C experiments in Δ3XCTCF, ΔPromGbx2 and Δ3XCTCF:ΔPromGbx2 mESC.*

Supplementary Figure 16. *RAD21 and H3K27ac profiles in ESC with genomic re-arrangements within the Gbx2/Asb18 locus.*

Supplementary Figure 17. *Genotyping of the Δ6XCTCF, 156Kb INV and Δ6XCTCF:156Kb INV re-arrangements generated at the Six3/Six2 locus.*

Supplementary Figure 18. *Genotyping of the 226Kb INV ESC lines.*

Supplementary Figure 19. *Genotyping of the Δ6XCTCF:Six2$^{-/-}$ ESC lines.*

Supplementary Figure 20. *Genotyping of the Six3$^{-/-}$ and Δ6XCTCF:Six3$^{-/-}$ deletions generated at the Six3/Six2 locus.*

Supplementary Figure 21. *Capture-C experiments in Δ6XCTCF, Six3$^{-/-}$ and Δ6XCTCF:Six3$^{-/-}$ NPC.*

Supplementary Figure 22. *RAD21 and H3K27ac profiles in NPC with genomic re-arrangements within the Six3/Six2 locus.*

Supplementary Figure 23. *Genotyping of the Δ4XCTCF and Δ4XCTCF:Six3$^{-/-}$ deletions generated at the Six3/Six2 locus.*

Supplementary References

Source Data

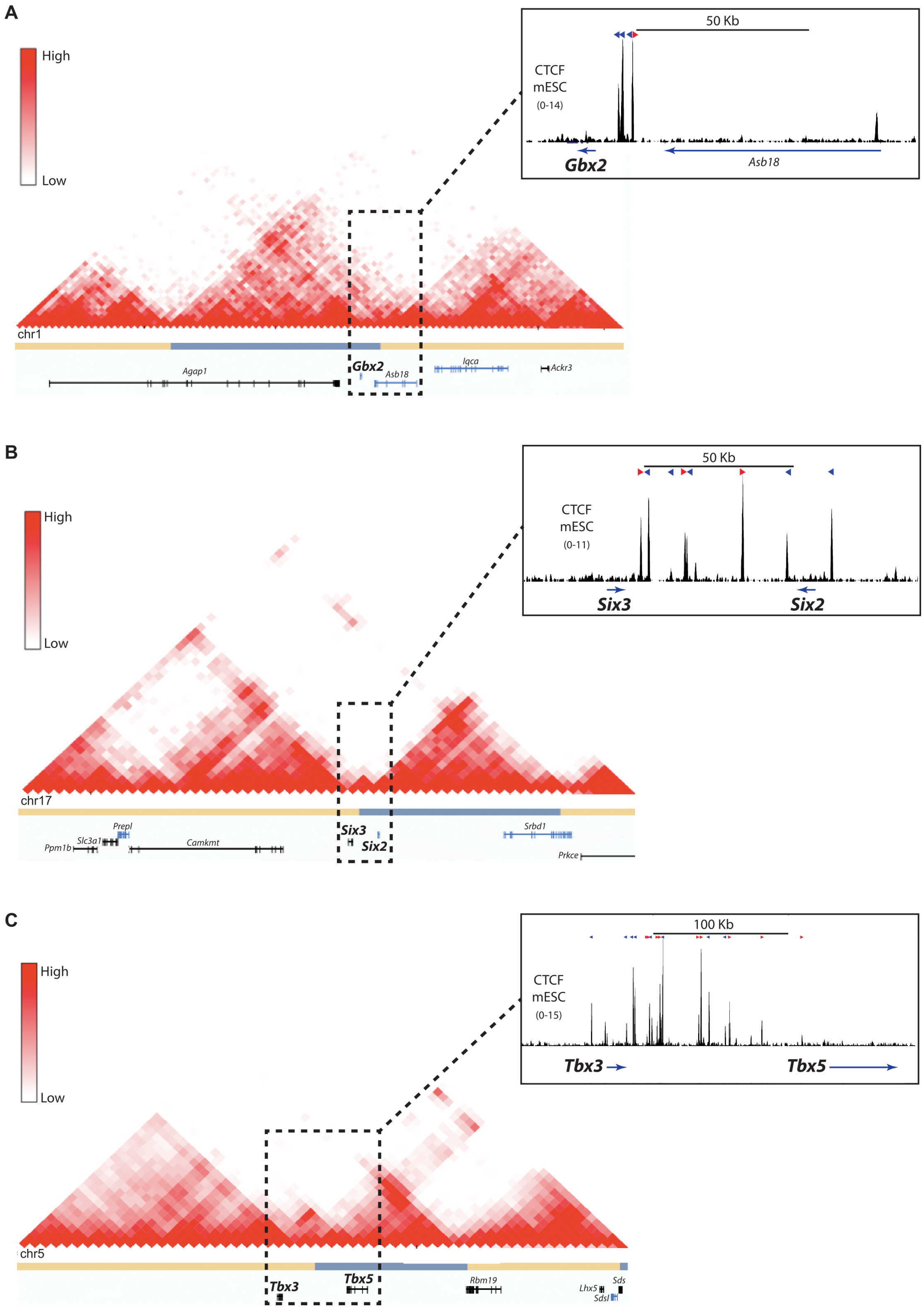

Supplementary Fig. 1: Hi-C profiles at representative examples of mouse developmental TADs. (A-C) *Hi-C data from mESC* [1] *around (A) Gbx2, (B) Six3/Six2 and (C) Tbx3/Tbx5, which serve as representative examples of mouse developmental genes located near TAD boundaries and within gene-poor TADs. CTCF ChIP-seq profiles in mESC* [2] *are shown around the TAD boundaries located near Gbx2, Six3/Six2 and Tbx3/Tbx5. The orientation of key CTCF sites is illustrated with red (sense) and blue (antisense) triangles.*

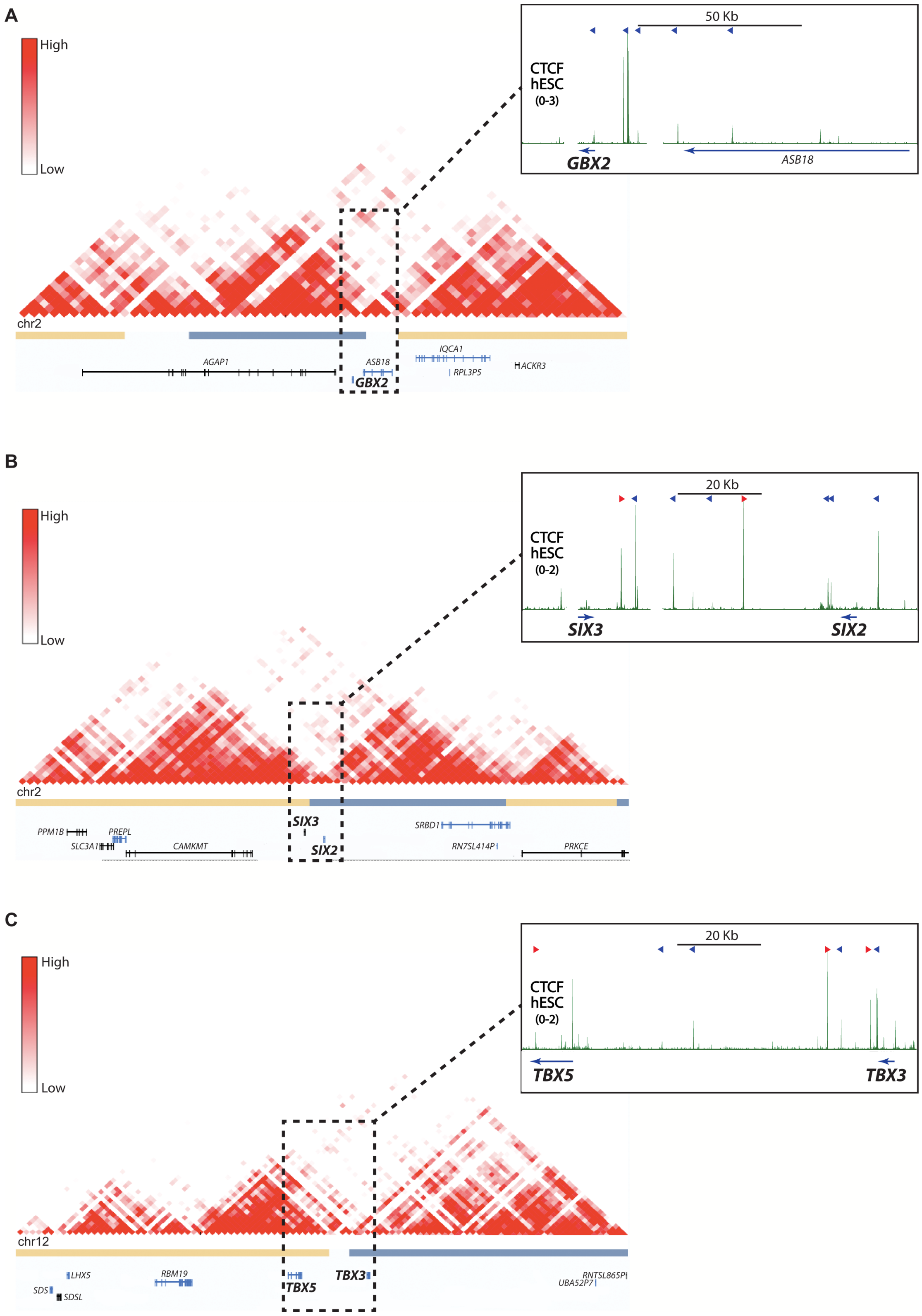

Supplementary Fig. 2: Hi-C profiles at representative examples of human developmental TADs. (A-C) *Hi-C data from hESC [3] around (A) GBX2, (B) SIX3/SIX2 and (C) TBX3/TBX5, which serve as representative examples of human developmental genes located near TAD boundaries and within gene-poor TADs. CTCF ChIP-seq profiles in hESC [2] are shown around the TAD boundaries located near GBX2, SIX3/SIX2 and TBX3/TBX5. The orientation of key CTCF sites is illustrated with red (sense) and blue (antisense) triangles.*

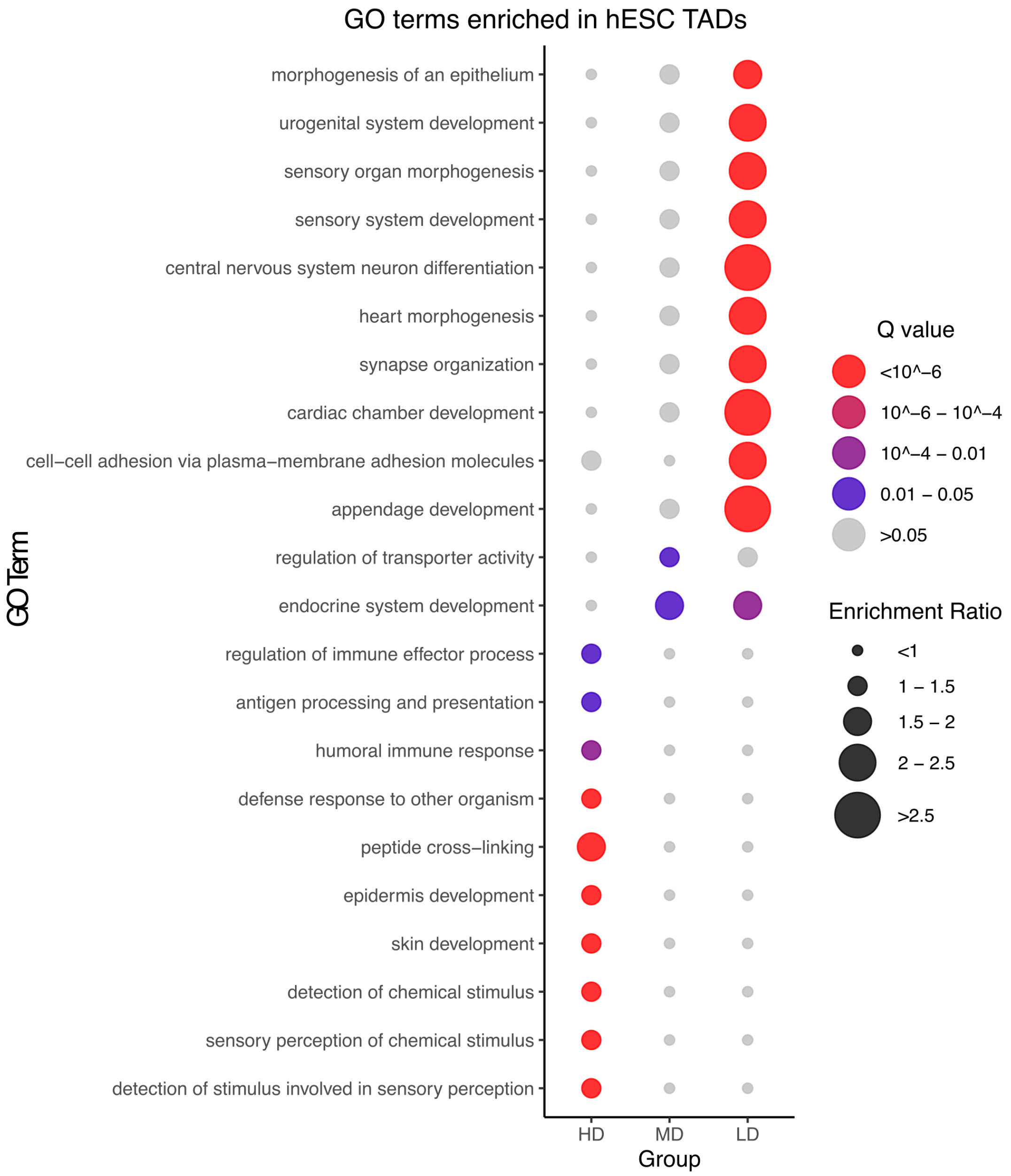

Supplementary Fig. 3: Enriched GO categories for human TADs with different gene densities. *TADs previously identified in hESC [3] were classified in three different groups based on their gene density: High Density (HD), Medium Density (MD) and Low Density (LD). Then, the genes present within each TAD group were subject to GO enrichment analysis. For the LD (n=130 enriched GO terms) group, only the top 10 most significantly enriched GO terms are highlighted (Q value ≤0.05), while for the MD (n=2 enriched GO terms) and HD (n=10 enriched GO terms) groups all the significantly enriched GO terms are presented.*

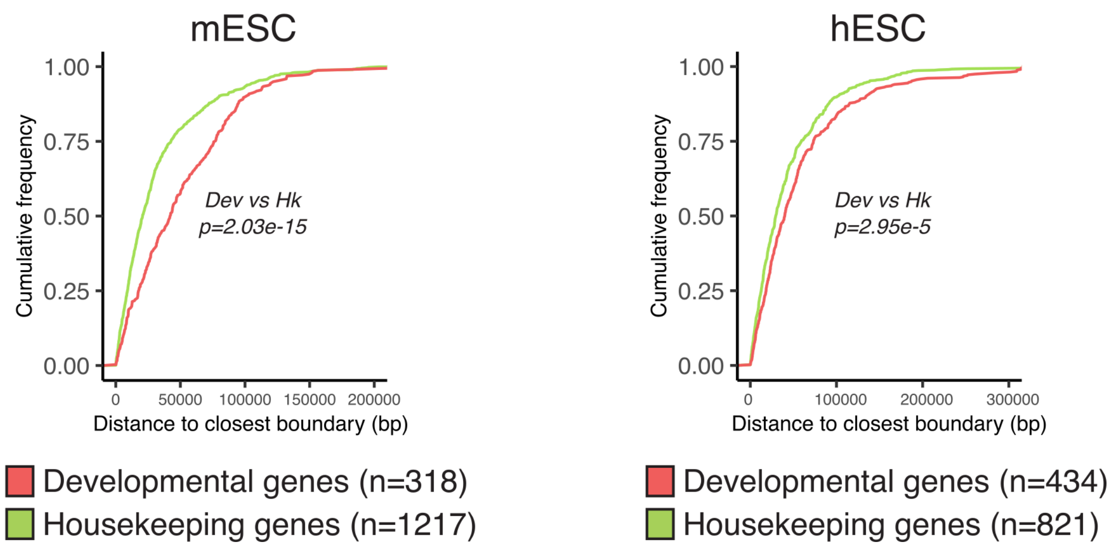

Supplementary Fig. 4: Boundary-proximal housekeeping genes are located at shorter distances from TAD boundaries than boundary-proximal developmental genes. *Developmental and housekeeping genes assigned to bin 1 (i.e. boundary-proximal) according to TAD maps generated in either mESC (left) or hESC (right) were considered (see Methods; Fig. 1B-C). Then, the cumulative distribution of the distances (in bp) separating either bin 1 developmental (red) or bin 1 housekeeping genes (green) from their nearest TAD boundary was calculated. P-values were calculated using unpaired two-sided Wilcoxon tests.*

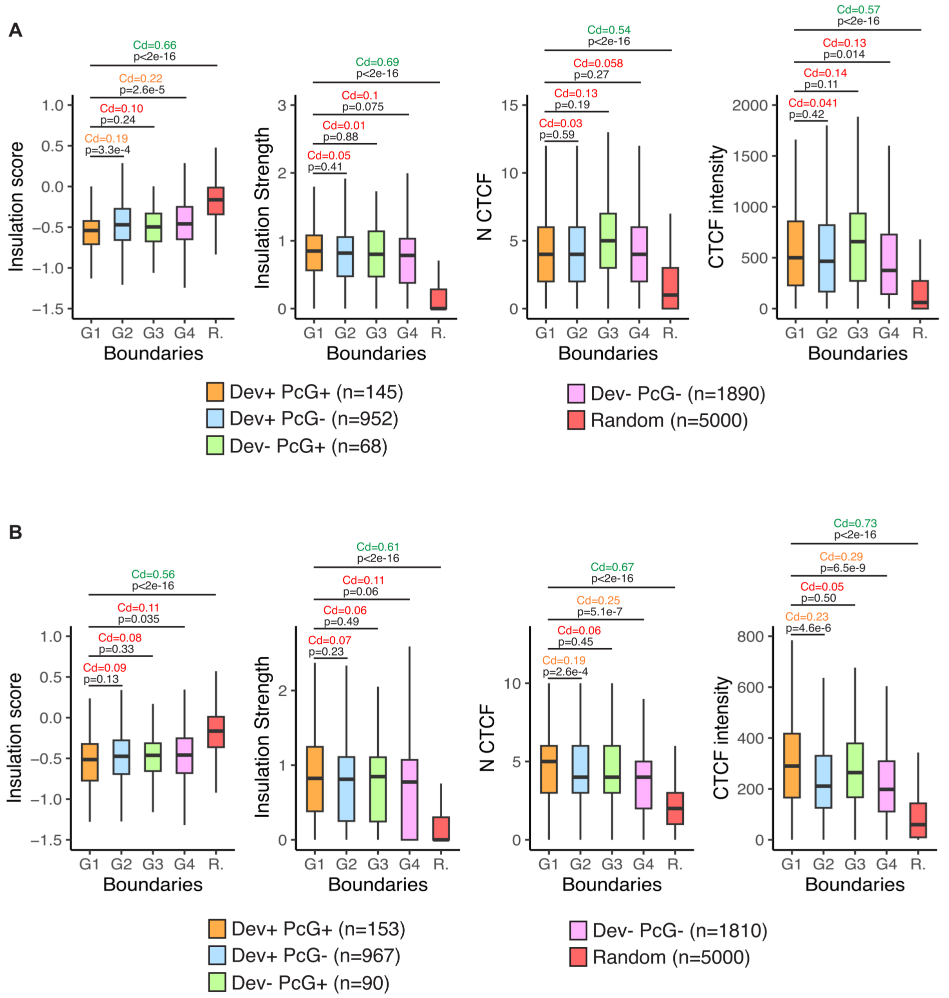

Supplementary Fig. 5: Insulation scores and boundary strength of TAD boundaries associated with bin 1 genes. (A-B) *TAD maps, Hi-C data and CTCF ChIP-seq profiles previously generated in either mESC (A) or hESC (B) were used to investigate the insulation scores, boundary strength, number of CTCF peaks and CTCF peaks aggregated signal at TAD boundaries associated with different types of bin 1 genes: Dev+PcG+(Developmental genes according to GO whose promoters are covered by broad PcG domains; n=145 for mESC and n=153 for hESC; orange), Dev+PcG- (Developmental genes according to GO whose promoters are not covered by broad PcG domains; n=952 for mESC and n=967 for hESC; blue), Dev-PcG+ (genes that are not considered as developmental in GO and whose promoters are covered by broad PcG domain; n=68 for mESC and n=90 for hESC; green); Dev-PcG- (genes that are not considered as developmental in GO and whose promoters are not covered by broad PcG domain; n=1890 for mESC and n=1810 for hESC; pink); random regions (n=5000; red). P-values were calculated using unpaired two-sided Wilcoxon tests with false discovery rate correction for multiple testing; Cliff's delta (Cd) effect sizes are shown as coloured numbers (green: large effect size; blue: medium effect size; orange: small effect size; red: negligible effect size). In the box plots, the upper and lower parts of the box are the upper and lower quartiles, respectively, the horizontal line that split the box in two is the median and the upper and lower whiskers indicate the maximum and minimum, respectively.*

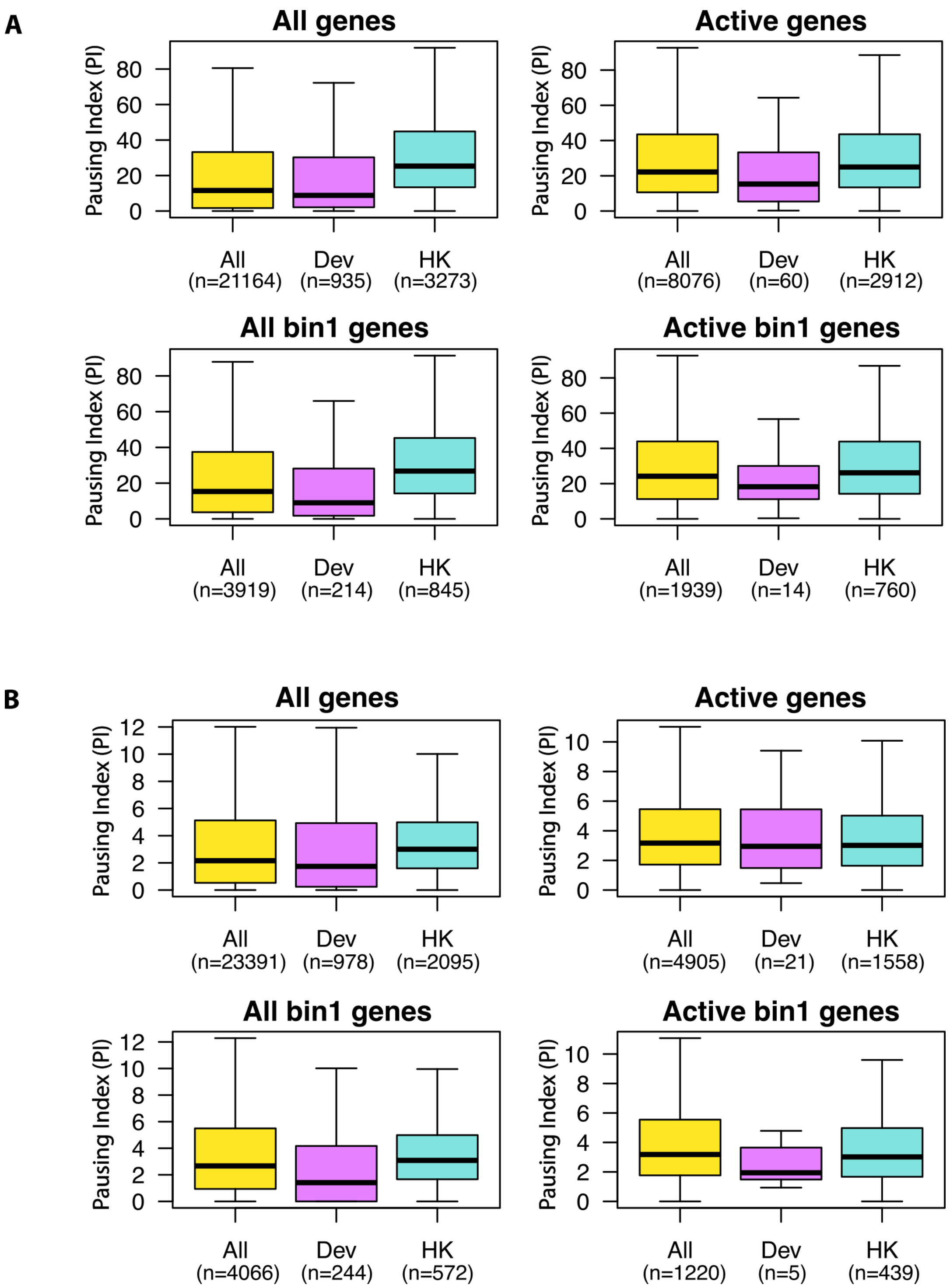

Supplementary Fig. 6: RNA Pol2 pausing index (PI) for different gene categories depending on their transcriptional status and proximity to TAD boundaries. (A-B) *PRO-seq data from mouse ESC (A) and GRO-seq data from human iPSC (B) (see Methods for details) was used to calculate the RNA Pol2 pausing index (PI) for different types of genes (i.e. housekeeping (green), developmental (purple), all (yellow)) according to their transcriptional status (Active genes: FPKM>5) and proximity to TAD boundaries (i.e. bin1 genes). In the box plots, the upper and lower parts of the box are the upper and lower quartiles, respectively, the horizontal line that split the box in two is the median and the upper and lower whiskers indicate the maximum and minimum, respectively.*

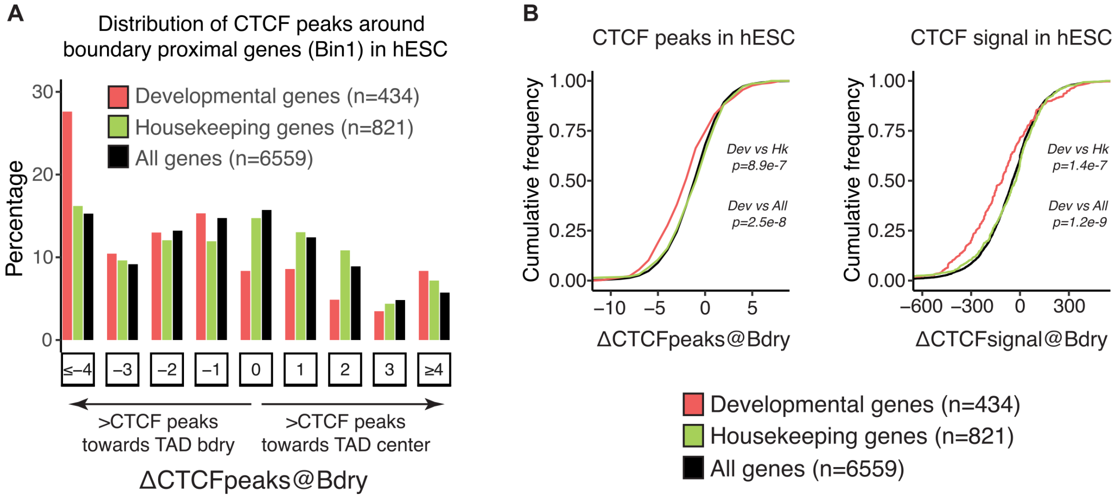

Supplementary Fig. 7: Developmental genes and clusters of CTCF sites are sequentially organized near human TAD boundaries. (A) *Histogram showing the distribution of ΔCTCFpeaks@Bdry values in hESC for different types of genes (developmental, housekeeping, all) located close to TAD boundaries (Bin 1 in Fig 1B).* ***(B)*** *Cumulative distribution plots for ΔCTCFpeaks@Bdry (left) and ΔCTCFsignal@Bdry (right) values in hESC show that for developmental genes the number of CTCF peaks and CTCF signals is significantly more skewed towards negative values than for the other considered gene categories. The ΔCTCFpeaks@Bdry and ΔCTCFsignal@Bdry metrics were calculated in hESC using previously reported TAD maps and CTCF ChIP-seq profiles (see Methods). P-values were calculated using unpaired two-sided Wilcoxon tests.*

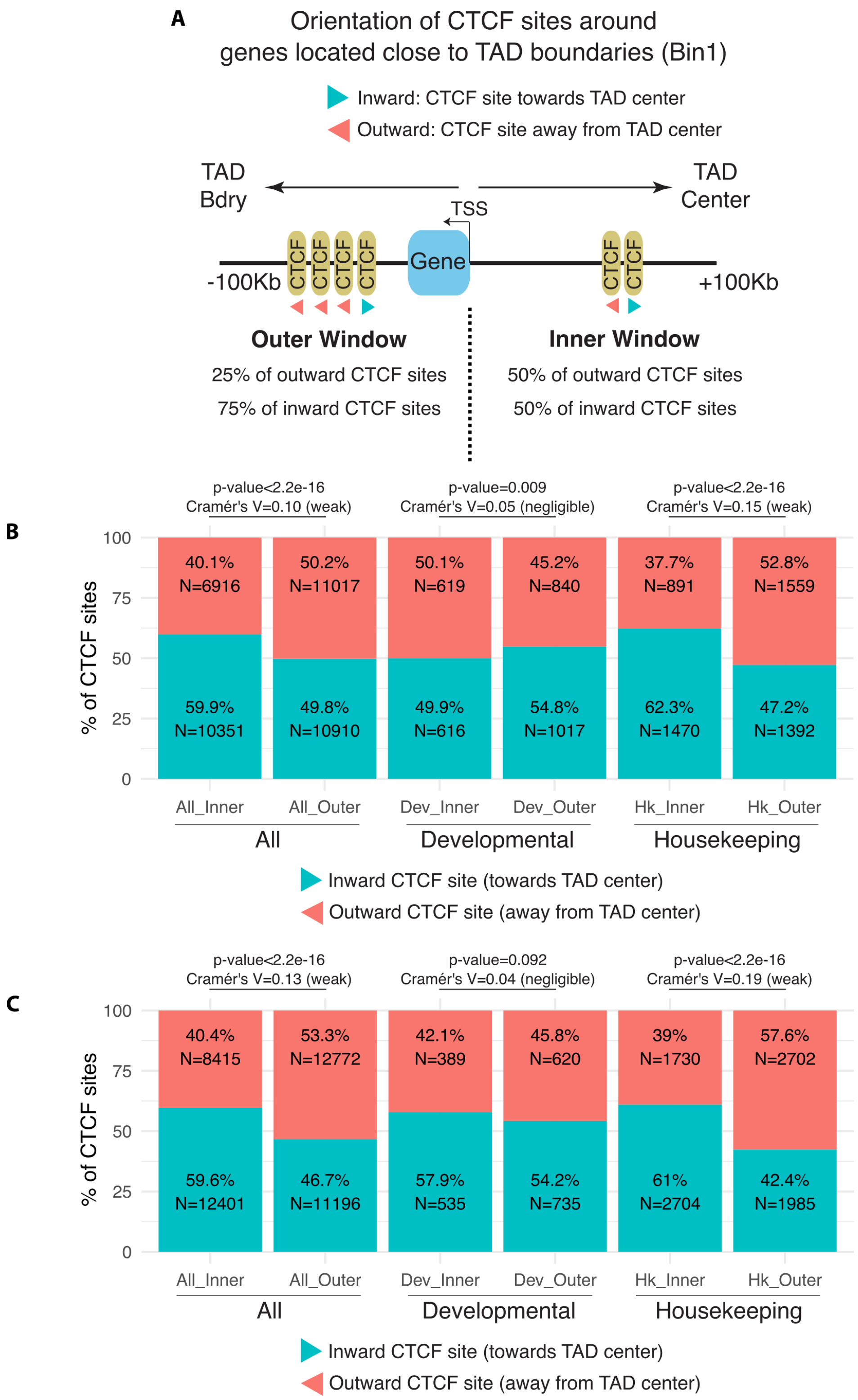

Supplementary Fig. 8: Orientation of CTCF sites around boundary-proximal genes. (A) *To investigate the orientation of CTCF sites around different types of genes (All, Developmental and Housekeeping) located close to TAD boundaries (i.e. bin 1 genes), the TSS of each gene was used as reference point considering a window of +/-100 Kb. Then, CTCF peaks located between the TSS and the TAD centers (Inner window) as well as CTCF peaks located between the TSS and the TAD boundaries (Outer window) were identified (see Methods for details regarding the TAD maps and CTCF ChIP-seq profiles used and that were*

previously generated in mESC and hESC). Finally, the orientation of the CTCF motifs associated with either Inner or Outer peaks with respect to the TAD center and the nearest boundary were calculated (CTCF sites oriented towards the TAD center are shown in blue and CTCF sites oriented towards the boundary are shown in red). ***(B-C)*** *The % of CTCF sites oriented towards either TAD centers or TAD boundaries are shown for human ESC (B) and mouse ESC (C) CTCF peaks located within the Inner and Outer windows described in (A). The total number of CTCF sites (N) are also indicated for each window and gene category. The p-values were calculated using Chi-square tests in order to evaluate whether there were significant differences in the distribution of the orientation of the CTCF sites between the Inner and Outer windows for each gene category (i.e. All, Developmental and Housekeeping). Effect sizes were calculated with Cramér's V estimator* [4]*.*

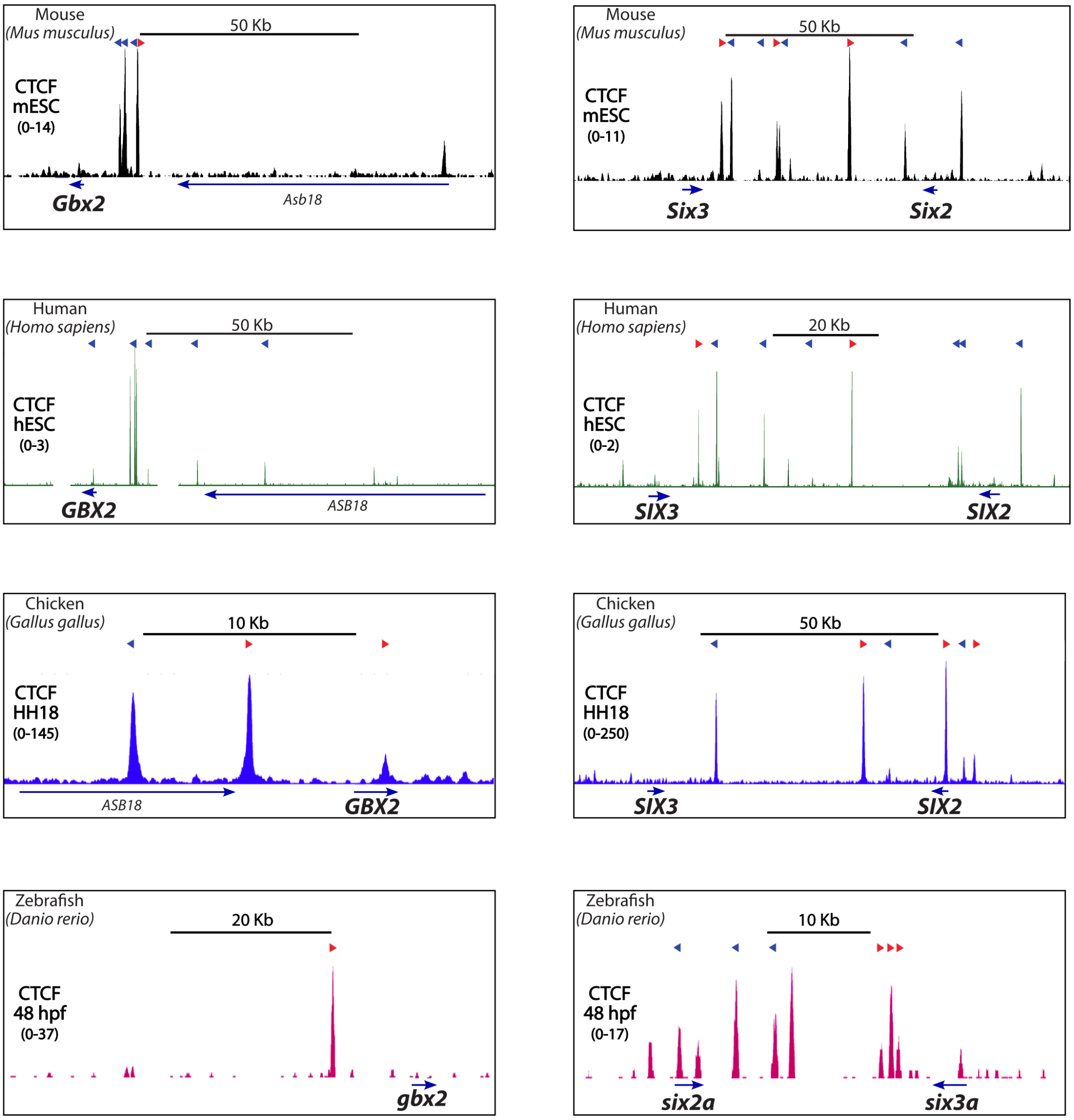

Supplementary Fig. 9: Conservation of CTFC clusters around the GBX2 and SIX3/SIX2 loci across vertebrates. *CTCF ChIP-seq profiles generated in mESC [2] , hESC [2], chicken embryos [5] and zebrafish embryos [6] are shown around GBX2 (left panel) and SIX3/SIX2 (right panels). In zebrafish, gbx2 and asb18 are located in different chromosomes. The orientation of CTCF sites is illustrated with red (sense) and blue (antisense) triangles.*

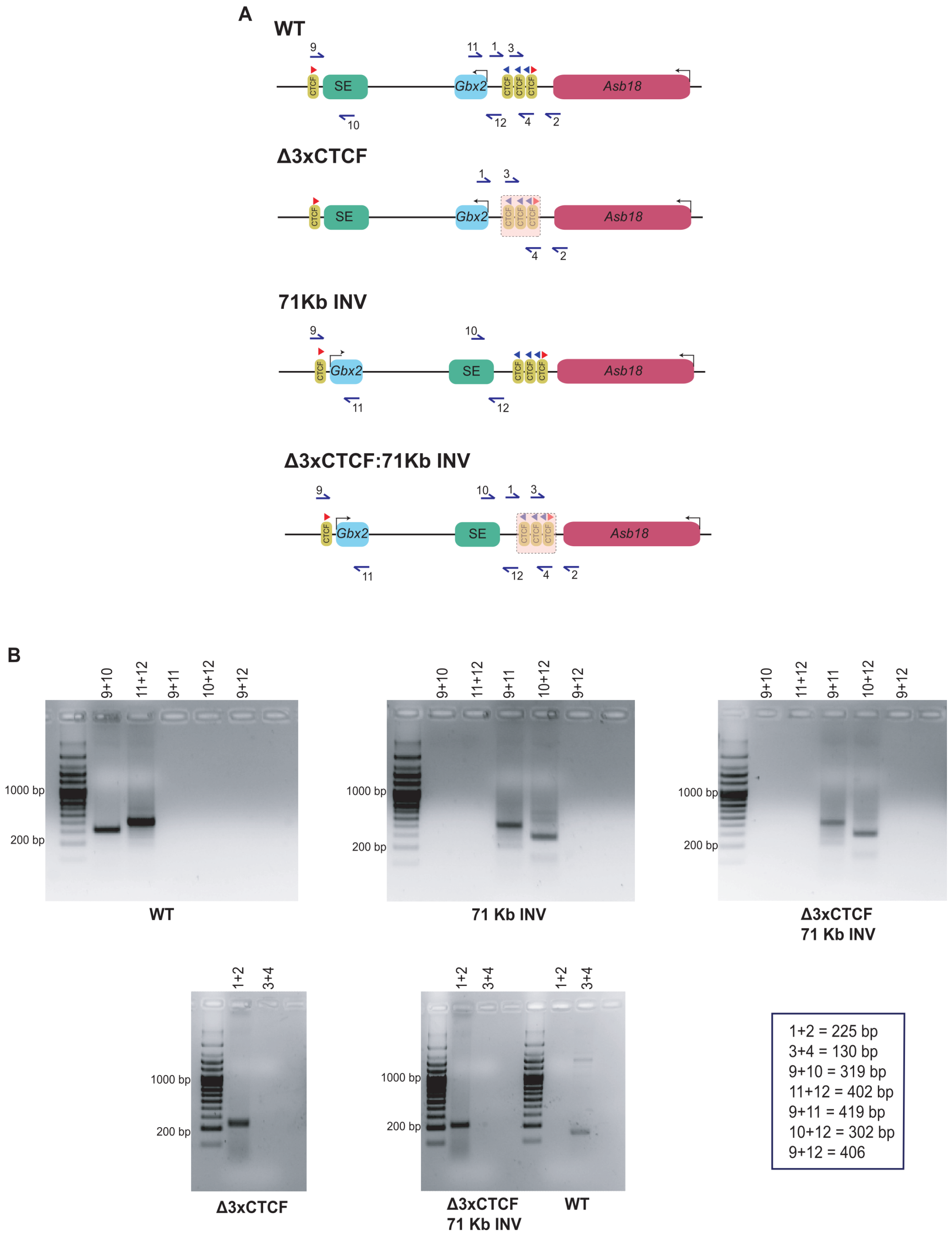

Supplementary Fig. 10: Genotyping of the Δ3XCTCF, 71Kb INV and Δ3XCTCF:71Kb INV re-arrangements generated at the Gbx2/Asb18 locus. (A) *Graphical overview of the PCR-based strategy used to genotype the Δ3XCTCF, 71Kb INV and Δ3XCTCF:71Kb INV genomic re-arrangements described in Fig 3. The horizontal arrows and accompanying numbers represent PCR primers.* ***(B)*** *Representative PCR genotyping results obtained for ESC lines that were either WT or homozygous for the Δ3XCTCF, 71 KB INV and Δ3XCTCF:71Kb INV genomic re-arrangements using the indicated primer pair combinations. The expected sizes of the amplicons obtained with each PCR primer combination are shown at the bottom right corner.*

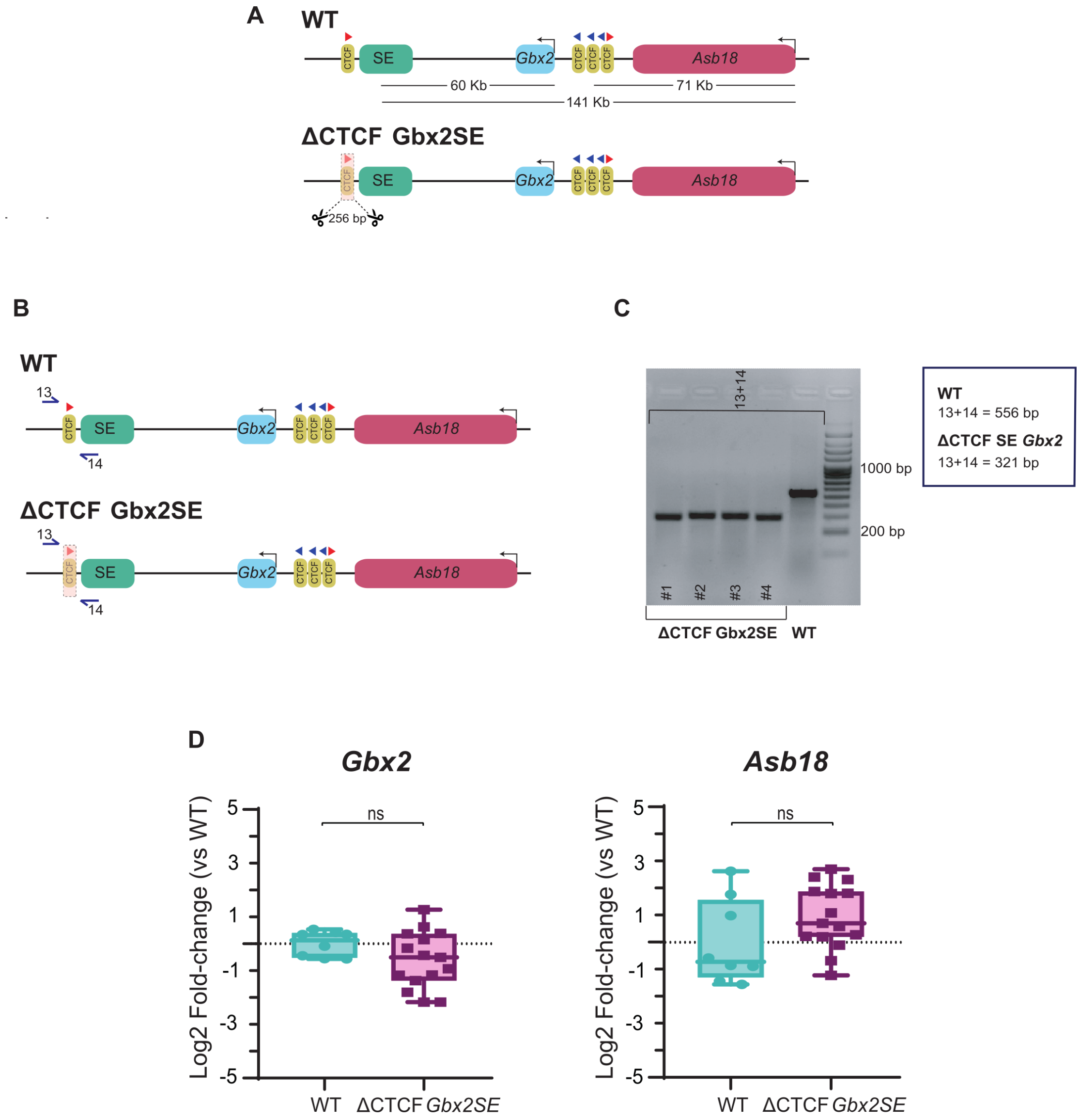

Supplementary Fig. 11: Assessing the role of the CTCF site located next to the Gbx2 SE in the control of Gbx2 expression. (A) *Graphical overview of the 256 bp deletion generated in mESC to eliminate the CTCF binding site located close to the Gbx2 SE (i.e. ΔCTCF Gbx2SE). The orientation of the CTCF sites are indicated with red (sense) and blue (antisense) triangles.* ***(B)*** *Graphical overview of the PCR-based strategy used to genotype the ΔCTCF Gbx2SE deletion described in (A). The horizontal blue arrows and accompanying numbers represent PCR primers.* ***(C)*** *Representative PCR genotyping results obtained for ESC lines that were either WT or homozygous for the ΔCTCF Gbx2SE deletion using the indicated primer pair combinations. The expected sizes of the amplicons obtained with each PCR primer combination are shown to the right.* ***(D)*** *The expression of Gbx2 and Asb18 was measured by RT–qPCR in mESC that were either WT or homozygous for the ΔCTCF Gbx2SE deletion. For each cell line, Gbx2 and Asb18 expression was measured in the following number of biological replicates: WT: 8 replicates; ΔCTCF Gbx2SE: 15 replicates using four different clonal lines. Expression values were normalized to two housekeeping genes (Eef1a1 and Hprt1) and are presented as log2 fold-changes with respect to WT ESCs.*

Expression differences among ESC lines were calculated using two-sided unpaired t-tests (NS (not significant) fold-change < 2 or P > 0.05). In (D) box plots, the upper and lower parts of the box are the upper and lower quartiles, respectively, the horizontal line that split the box in two is the median and the upper and lower whiskers indicate the maximum and minimum, respectively. Source RT-qPCR data are provided as a Source Data file.

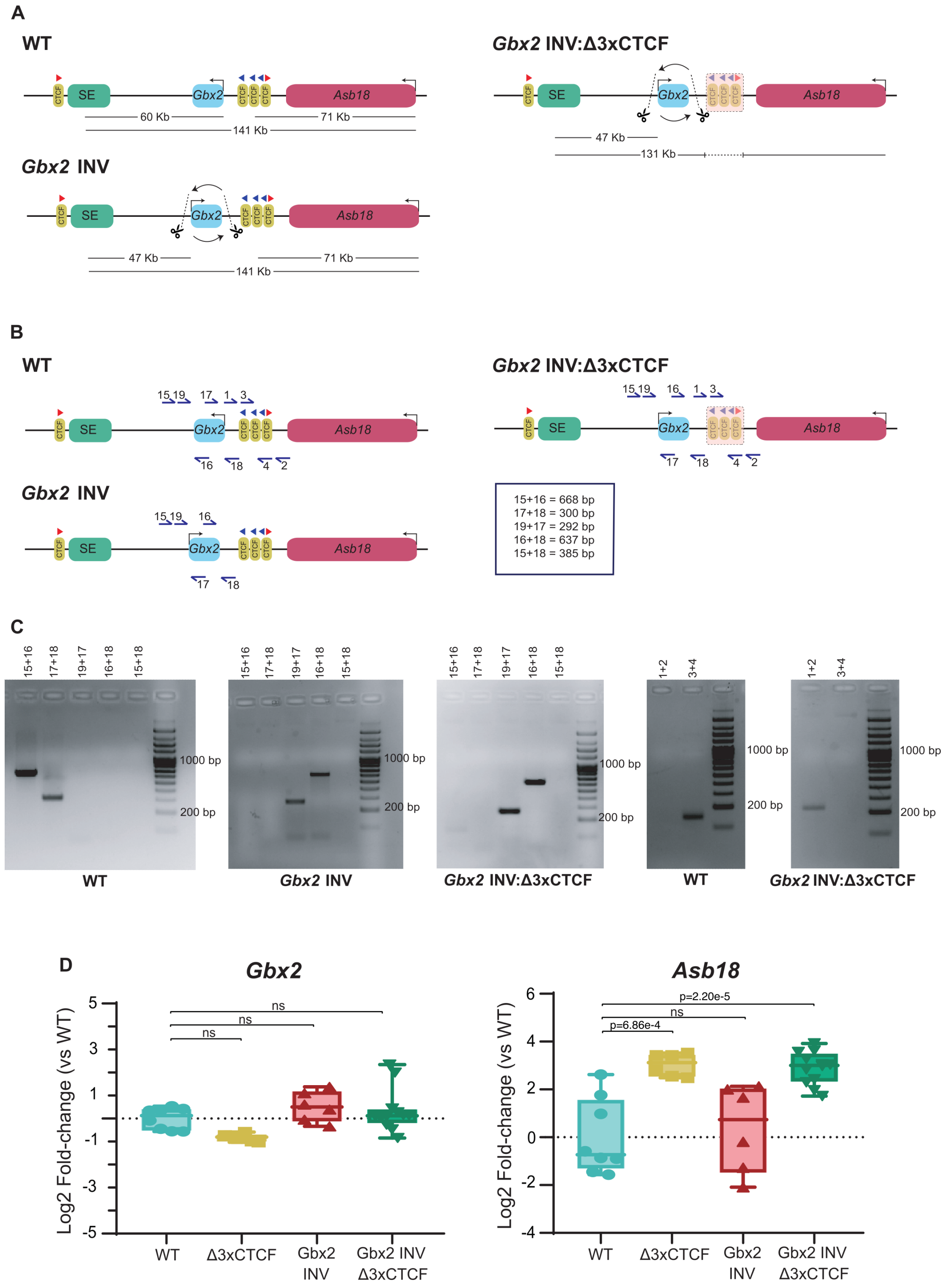

Supplementary Fig. 12: The orientation of Gbx2 does not have a major role in the insulation of its own regulatory domain. (A) *Graphical overview of the following genomic rearrangements generated in mESC within the Gbx2/Asb18 locus: Gbx2 INV – 18 kb inversion spanning the whole Gbx2 gene; Gbx2 INV:Δ3xCTCF – combination of the 18 kb inversion described above and 10 kb deletion that eliminates the three CTCF sites separating the Gbx2-TAD and Asb18-TAD (Fig. 3). The effects of these re-arrangements on the linear distances separating Gbx2, Asb18 and the SE are indicated. The orientation of the CTCF*

sites are indicated with red (sense) and blue (antisense) triangles. ***(B)*** *Graphical overview of the PCR-based strategy used to genotype the Gbx2 INV and Gbx2 INV:Δ3xCTCF genomic re-arrangements described in (A). The horizontal blue arrows and accompanying numbers represent PCR primers. The expected sizes of the amplicons obtained with each PCR primer combination are shown at the bottom right corner.* ***(C)*** *Representative PCR genotyping results obtained for ESC lines that were either WT, homozygous for Gbx2 INV or homozygous for Gbx2 INV:Δ3xCTCF using the indicated primer pair combinations.* ***(D)*** *The expression of Gbx2 and Asb18 was measured by RT–qPCR in ESCs that were either WT or homozygous for the genomic re-arrangements described in (A). For each cell line, Gbx2 and Asb18 expression was measured in the following number of biological replicates: WT: 8 replicates; Δ3XCTCF: 6 replicates using two different clonal lines; Gbx2 INV: 6 replicates using one clonal line; Gbx2 INV:Δ3xCTCF: 12 replicates using two different clonal lines. Expression values were normalized to two housekeeping genes (Eef1a1 and Hprt1) and are presented as log2 fold-changes with respect to WT ESCs. Expression differences among ESC lines were calculated using two-sided unpaired t-tests (***fold-change > 2 and P < 0.001; NS (not significant) fold-change < 2 or P > 0.05). In (D) box plots, the upper and lower parts of the box are the upper and lower quartiles, respectively, the horizontal line that split the box in two is the median and the upper and lower whiskers indicate the maximum and minimum, respectively.Source RT-qPCR data are provided as a Source Data file.*

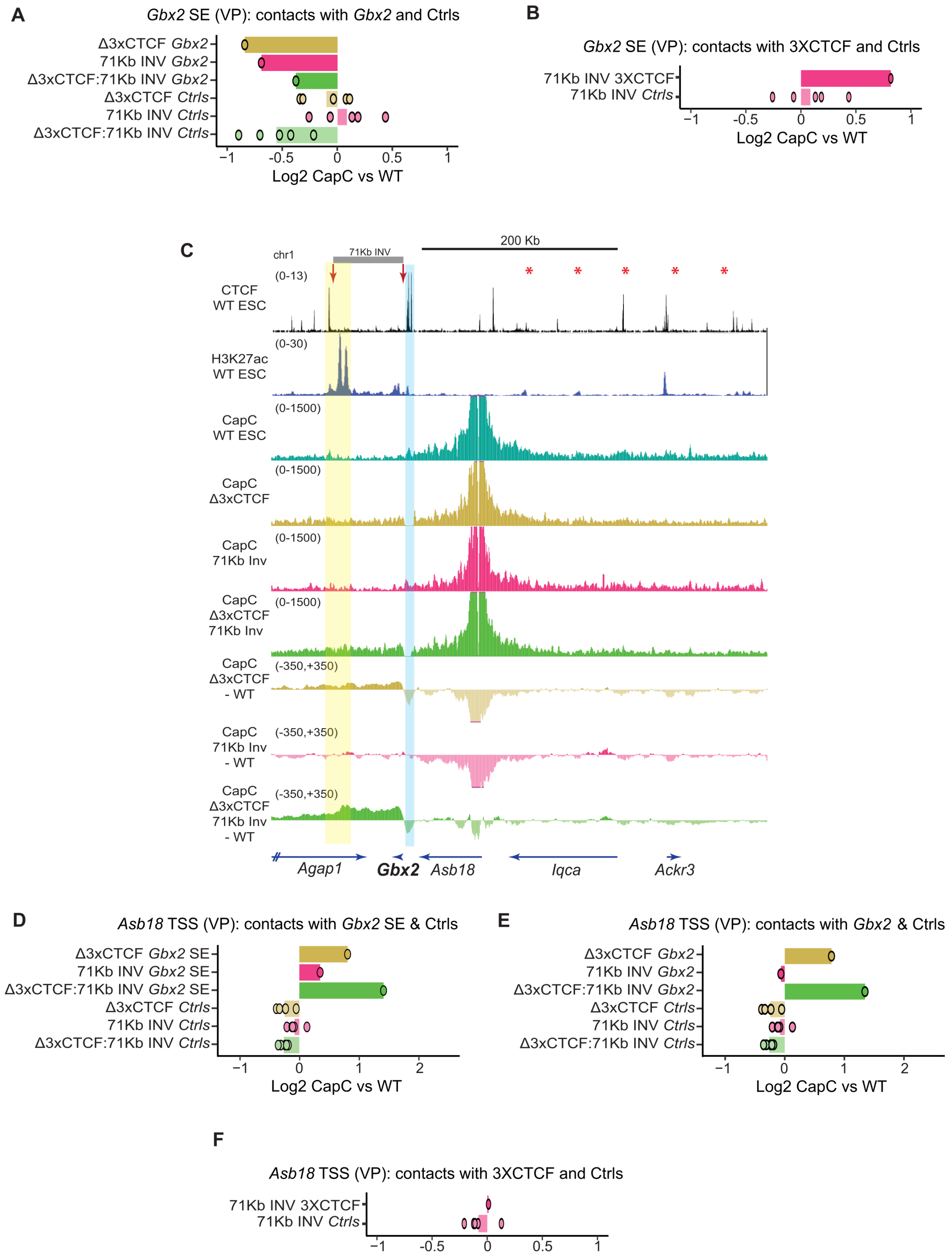

Supplementary Fig. 13: Capture-C experiments in Δ3XCTCF, 71Kb INV and Δ3XCTCF:71Kb INV mESC. (A-B) *The average Capture-C signals shown in Fig. 3E (i.e. using the Gbx2 SE as a viewpoint) were measured within (A) the Gbx2 gene (chr1:89922961-89936176 (mm10)), (B) the 3XCTCF cluster (chr1:89938921-89943877 (mm10)) and within five different 30 Kb control regions (Ctrls) located within the Gbx2 TAD (red asterisks in Fig.3E indicate the midpoint of the following controls regions (mm10): chr1:89803398-89833398; chr1:89753398-89783398; chr1:89703398-89733398; chr1:89653398-89683398;*

chr1:89603398-89633398). ***(C)*** *Capture-C experiments were performed as two biological replicates in WT, Δ3XCTCF, 71Kb INV and Δ3XCTCF:71Kb INV ESC using the Asb18 promoter as a viewpoint. The average Capture-C signals of the two replicates performed for each mESC line are shown around the Gbx2/Asb18 locus either individually (upper tracks) or after subtracting the WT Capture-C signals (lower tracks), using in all cases the mm10 reference genome. The TAD boundary containing the three CTCF sites is highlighted in light blue. The red arrows indicate the 71 Kb inversion breakpoints. (****D-F****) The average Capture-C signals shown in (C) were measured around (D) the Gbx2 SE (highlighted in yellow in (C); chr1:89858398-89889044 (mm10)), (E) the Gbx2 gene (chr1:89922961-89936176 (mm10)), (F) the 3XCTCF cluster (highlighted in blue in (C); chr1:89938921-89943877 (mm10)) and within five different 30 Kb control regions (Ctrls) located within the Asb18 TAD (red asterisks in (C) indicate the midpoint of the following controls regions (mm10): chr1:90049577-90079577, chr1:90099577-90129577, chr1:90149577-90179577, chr1:90199577-90229577, chr1:90249577-90279577). In (A-B) and (D-F), Capture-C signals are shown for the Δ3XCTCF, 71Kb INV and Δ3XCTCF:71Kb INV ESC as log2 fold-changes with respect to WT ESC. In (B) and (F), the Capture-C signals were not measured around the 3XCTCF cluster in those ESC lines in which that region was deleted (i.e. Δ3XCTCF and Δ3XCTCF:71Kb INV).*

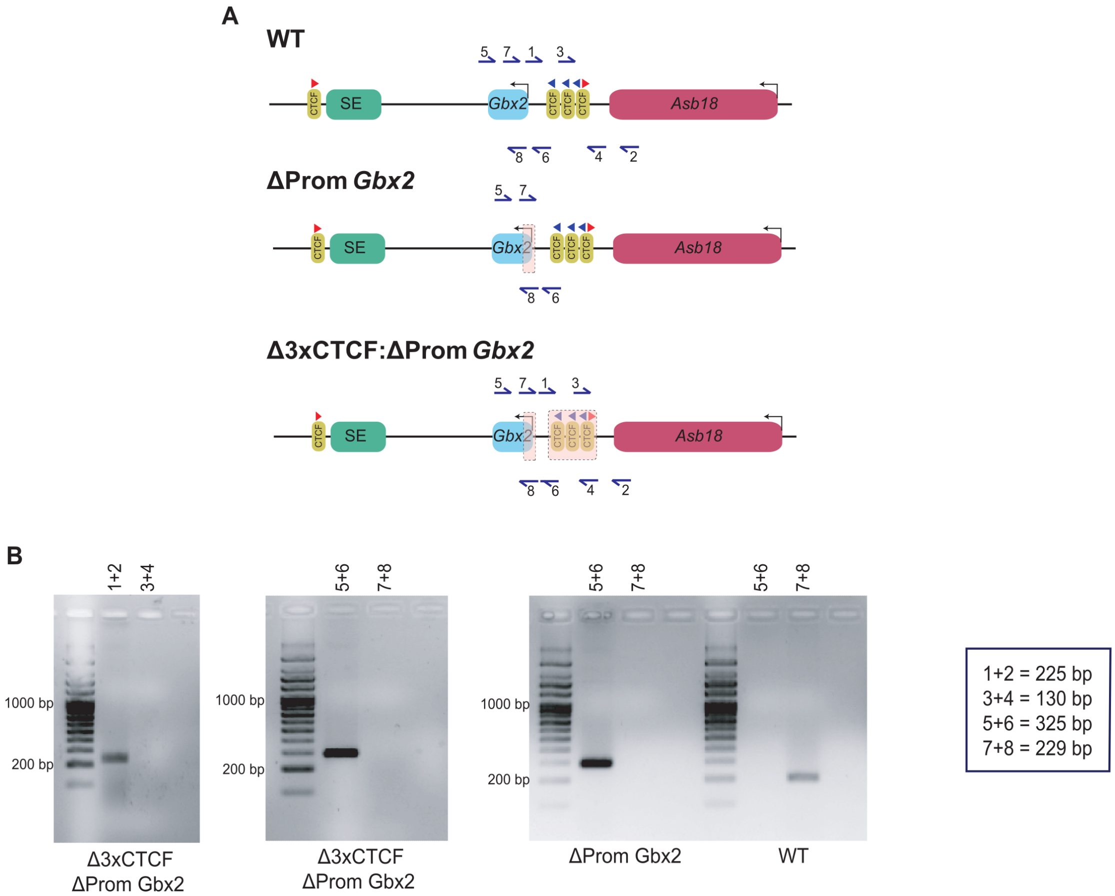

Supplementary Fig. 14: Genotyping of the ΔPromGbx2 and Δ3XCTCF:ΔPromGbx2 re-arrangements generated at the Gbx2/Asb18 locus. (A) *Graphical overview of the PCR-based strategy used to genotype the ΔPromGbx2 and Δ3XCTCF:ΔPromGbx2 deletions described in Fig 4. The horizontal arrows and accompanying numbers represent PCR primers.* ***(B)*** *Representative PCR genotyping results obtained for ESC lines that were either WT or homozygous for the ΔPromGbx2 and Δ3XCTCF:ΔPromGbx2 deletions using the indicated primer pair combinations. The expected sizes of the amplicons obtained with each PCR primer combination are shown at the bottom right corner.*

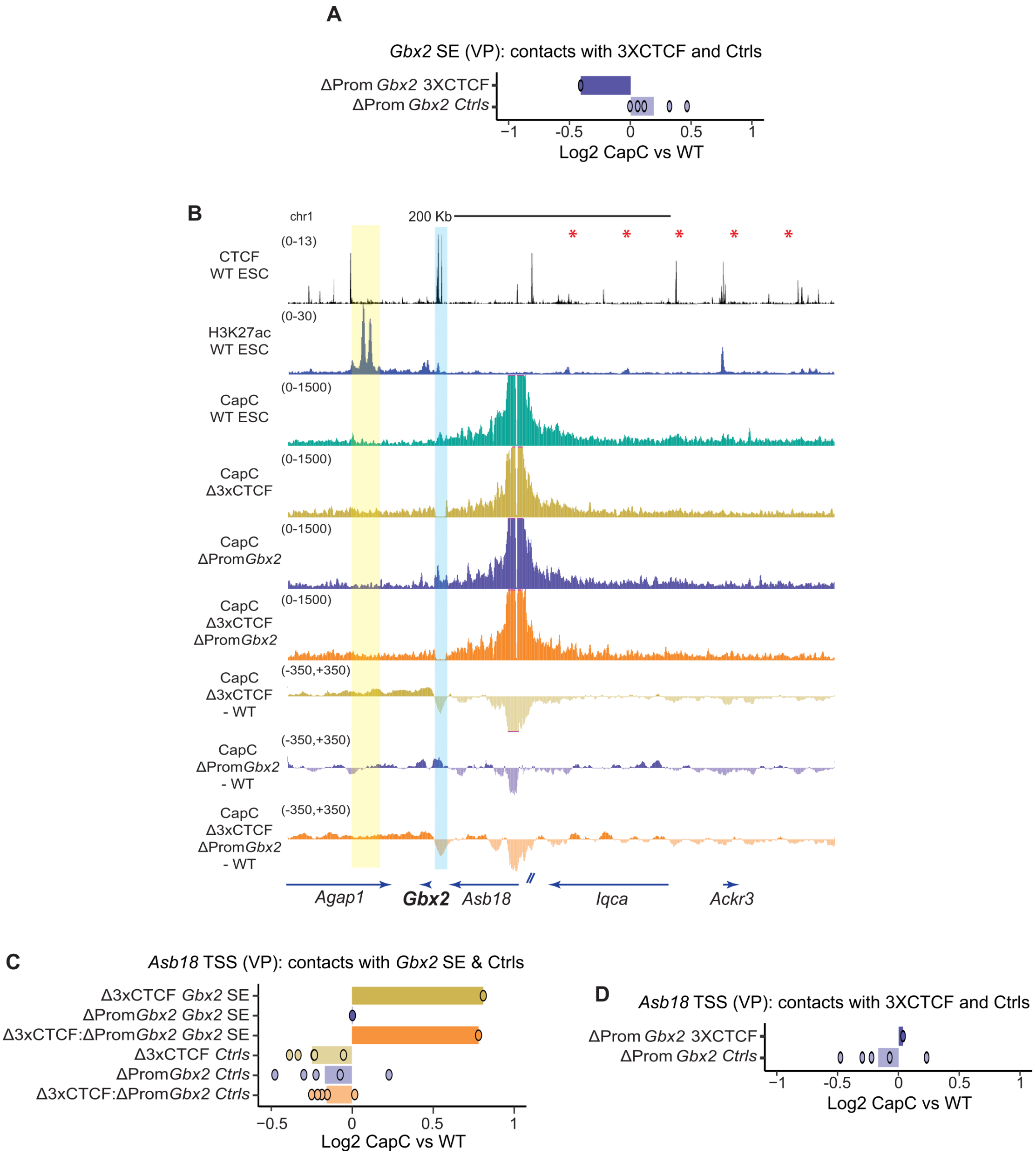

Supplementary Fig. 15: Capture-C experiments in Δ3XCTCF, ΔPromGbx2 and Δ3XCTCF:ΔPromGbx2 mESC. (A) *The average Capture-C signals shown in Fig. 4D (i.e. using the Gbx2 SE as a viewpoint) were measured within the 3XCTCF cluster (chr1:89938921-89943877 (mm10)) and within five different 30 Kb control regions (Ctrls) located within the Gbx2 TAD (red asterisks in Fig.4D indicate the midpoint of the following controls regions (mm10): chr1:89803398-89833398; chr1:89753398-89783398; chr1:89703398-89733398; chr1:89653398-89683398; chr1:89603398-89633398).* ***(B)*** *Capture-C experiments were performed as two biological replicates in WT, Δ3XCTCF, ΔPromGbx2 and Δ3XCTCF:ΔPromGbx2 ESC using the Asb18 promoter as a viewpoint. The average Capture-C signals of two replicates for each mESC line are shown around the Gbx2/Asb18 locus either individually (upper tracks) or after subtracting the WT Capture-C signals (lower tracks), using in all cases the mm10 reference genome. The TAD boundary containing the three CTCF sites is highlighted in light blue. (****C-D****) The average Capture-C*

signals shown in (B) were measured around the Gbx2 SE (highlighted in yellow in (B); chr1:89858398-89889044 (mm10)), the 3XCTCF cluster (highlighted in blue in (B); chr1:89938921-89943877 (mm10)) and within five different 30 Kb control regions (Ctrls) located within the Asb18 TAD (red asterisks in (B) indicate the midpoint of the same control regions as in Fig S11C-F). Capture-C signals are shown for the Δ3XCTCF, ΔPromGbx2 and Δ3XCTCF:ΔPromGbx2 ESC as log2 fold-changes with respect to WT ESC. In (A) and (D), the Capture-C signals were not measured around the 3XCTCF cluster in those ESC lines in which that region was deleted (i.e. Δ3XCTCF and Δ3XCTCF:ΔPromGbx2). Moreover, Capture-C signals around the Gbx2 gene were not measured in ESC lines in which the Gbx2 promoter was deleted (i.e. ΔPromGbx2 and Δ3XCTCF:ΔPromGbx2). See Fig. S11 for quantification of Capture-C signals around the Gbx2 gene in WT and Δ3XCTCF ESC.

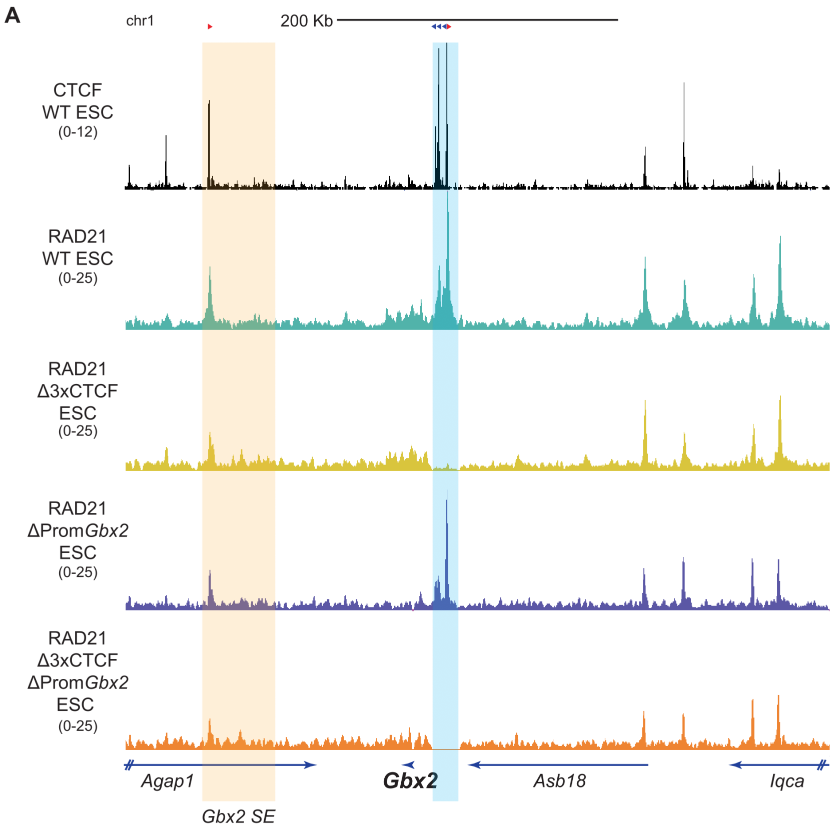

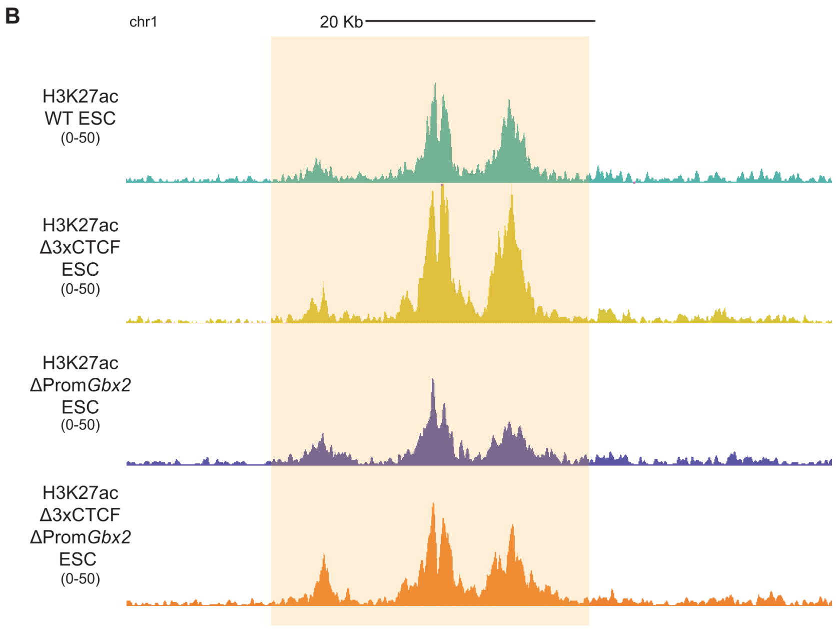

Supplementary Fig. 16: RAD21 and H3K27ac profiles in ESC with genomic re-arrangements within the Gbx2/Asb18 locus. (A-B) *ChIP-seq profiles for RAD21 (A) and H3K27ac (B) are shown in mESC that are either WT or homozygous for the Δ3XCTCF, ΔPromGbx2 or Δ3XCTCF:ΔPromGbx2 deletions. In (A), CTCF ChiP-seq profiles generated in WT mESC [2] are also shown on top. The Gbx2 SE is highlighted in orange and the three CTCF sites deleted within the Gbx2/Asb18 TAD boundary are highlighted in blue. The orientation of key CTCF sites is illustrated with red (sense) and blue (antisense) triangles.*

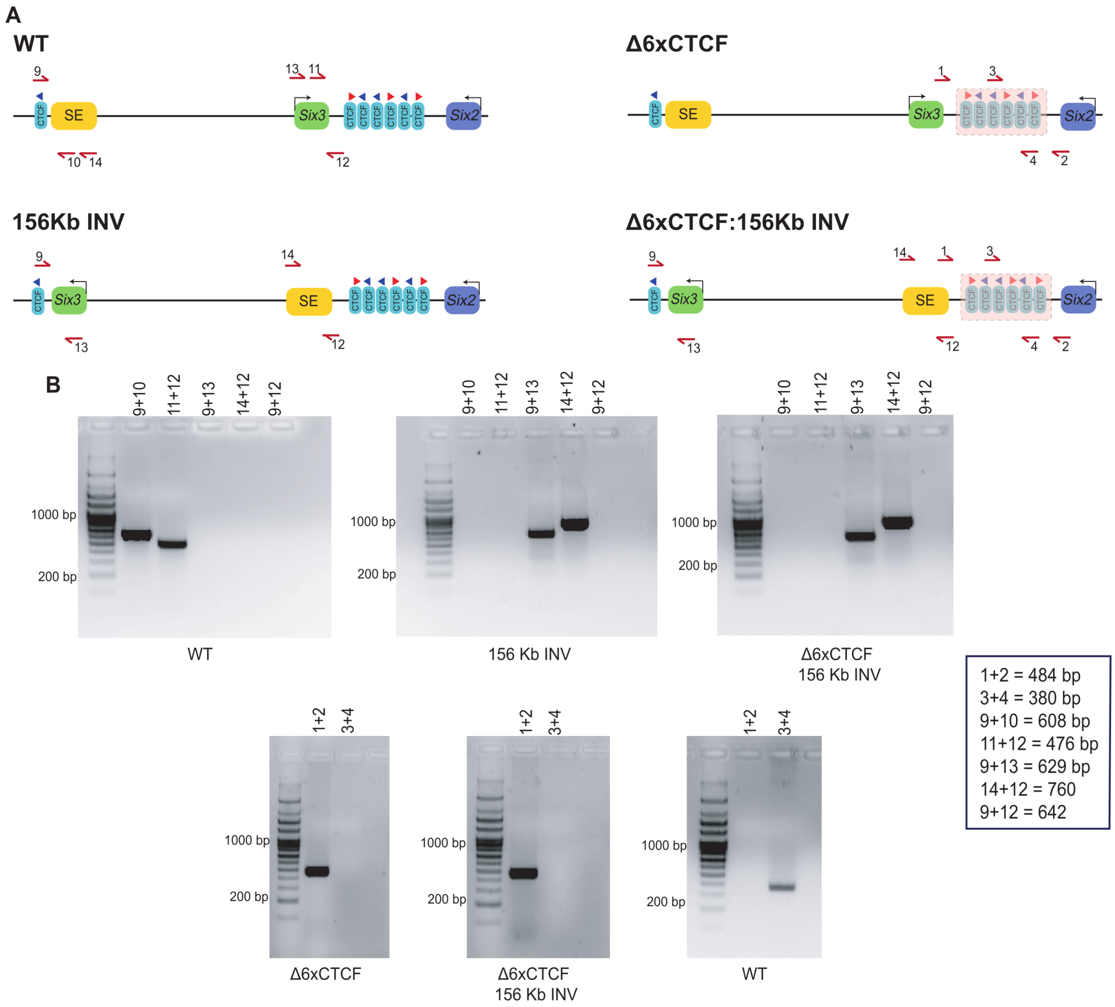

Supplementary Fig. 17: Genotyping of the Δ6XCTCF, 156Kb INV and Δ6XCTCF:156Kb INV re-arrangements generated at the Six3/Six2 locus. (A) *Graphical overview of the PCR-based strategy used to genotype the Δ6XCTCF, 156Kb INV and Δ3XCTCF:156Kb INV genomic re-arrangements described in Fig 5. The horizontal arrows and accompanying numbers represent PCR primers.* ***(B)*** *Representative PCR genotyping results obtained for ESC lines that were either WT or homozygous for the 6XCTCF, 156Kb INV and Δ6XCTCF:156Kb INV genomic re-arrangements using the indicated primer pair combinations. The expected sizes of the amplicons obtained with each PCR primer combination are shown at the bottom right corner.*

.

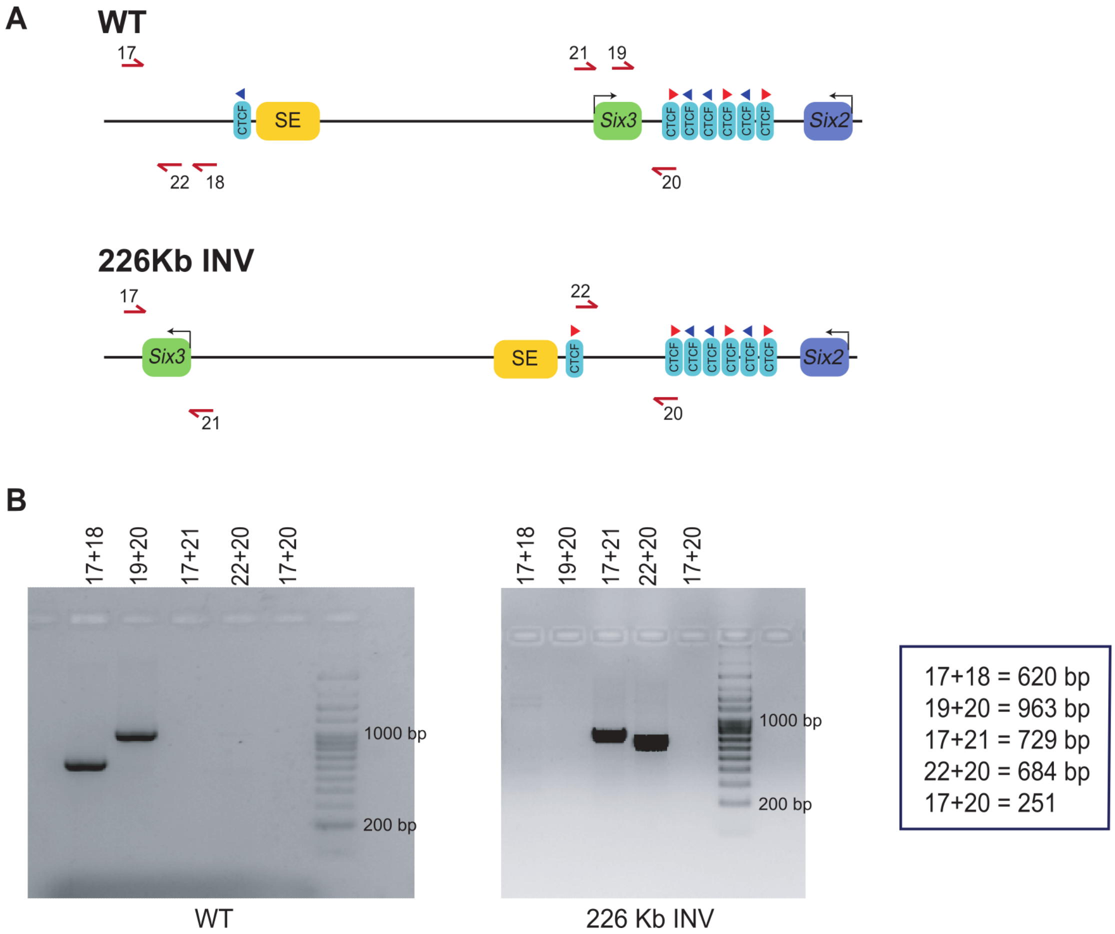

Supplementary Fig. 18: Genotyping of the 226Kb INV ESC lines. (A) *Graphical overview of the PCR-based strategy used to genotype the 226 Kb INV inversion described in Fig 5. The horizontal arrows and accompanying numbers represent PCR primers.* ***(B)*** *Representative PCR genotyping results obtained for ESC lines that were either WT or homozygous for the 226 Kb INV inversion using the indicated primer pair combinations. The expected sizes of the amplicons obtained with each PCR primer combination are shown at the bottom right corner.*

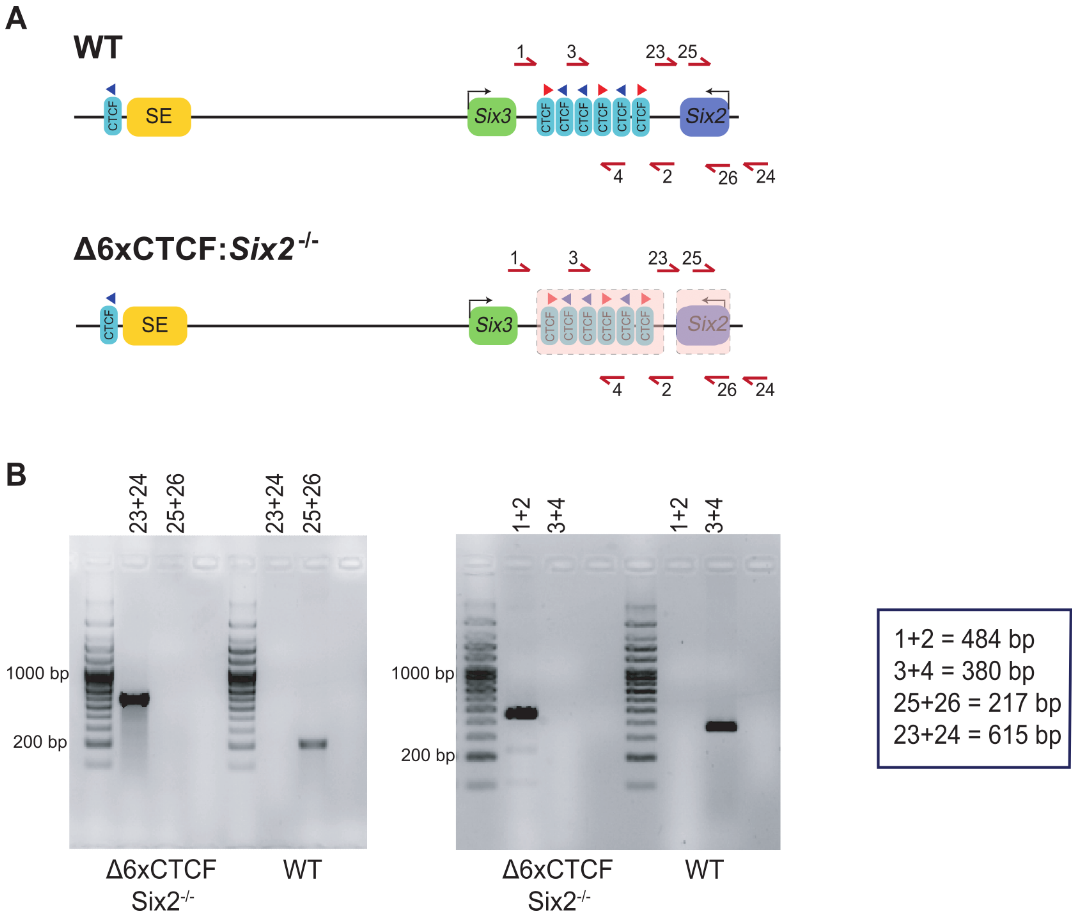

Supplementary Fig. 19: Genotyping of the Δ6XCTCF:Six2$^{-/-}$ ESC lines. (A) *Graphical overview of the PCR-based strategy used to genotype the Δ6XCTCF:Six2$^{-/-}$ deletions described in Fig 5. The horizontal arrows and accompanying numbers represent PCR primers.* ***(B)*** *Representative PCR genotyping results obtained for ESC lines that were either WT or homozygous for the Δ6XCTCF:Six2$^{-/-}$ deletions using the indicated primer pair combinations. The expected sizes of the amplicons obtained with each PCR primer combination are shown at the bottom right corner.*

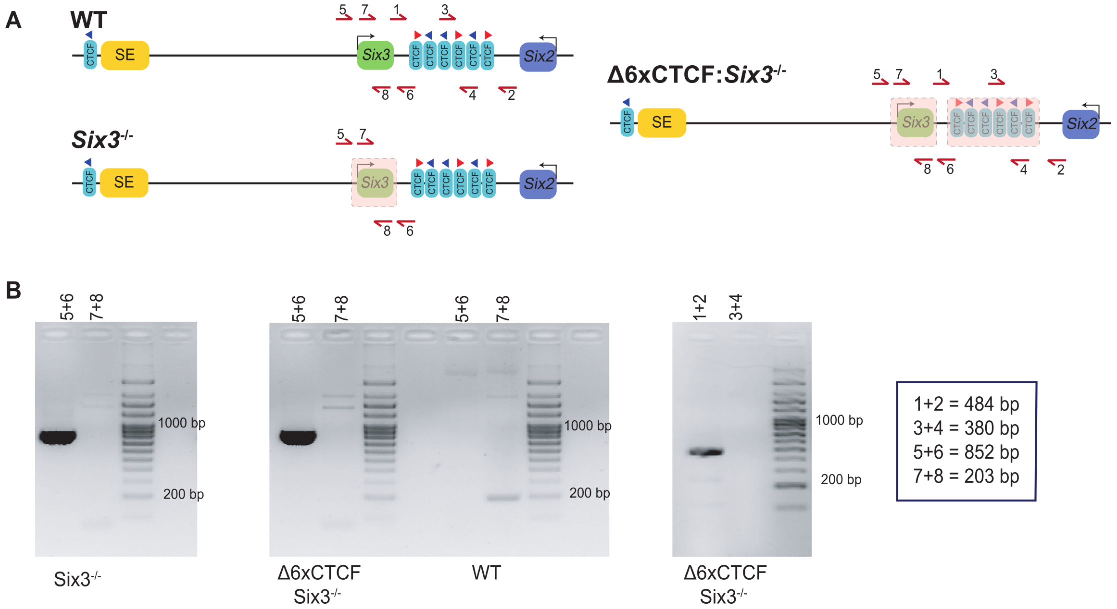

Supplementary Fig. 20: Genotyping of the Six3$^{-/-}$ and Δ6XCTCF:Six3$^{-/-}$ deletions generated at the Six3/Six2 locus. (A) *Graphical overview of the PCR-based strategy used to genotype the Six3$^{-/-}$ and Δ6XCTCF:Six3$^{-/-}$ deletions described in Fig 6. The horizontal arrows and accompanying numbers represent PCR primers.* ***(B)*** *Representative PCR genotyping results obtained for ESC lines that were either WT or homozygous for the Six3$^{-/-}$ and Δ6XCTCF:Six3$^{-/-}$ deletions using the indicated primer pair combinations. The expected sizes of the amplicons obtained with each PCR primer combination are shown at the bottom right corner.*

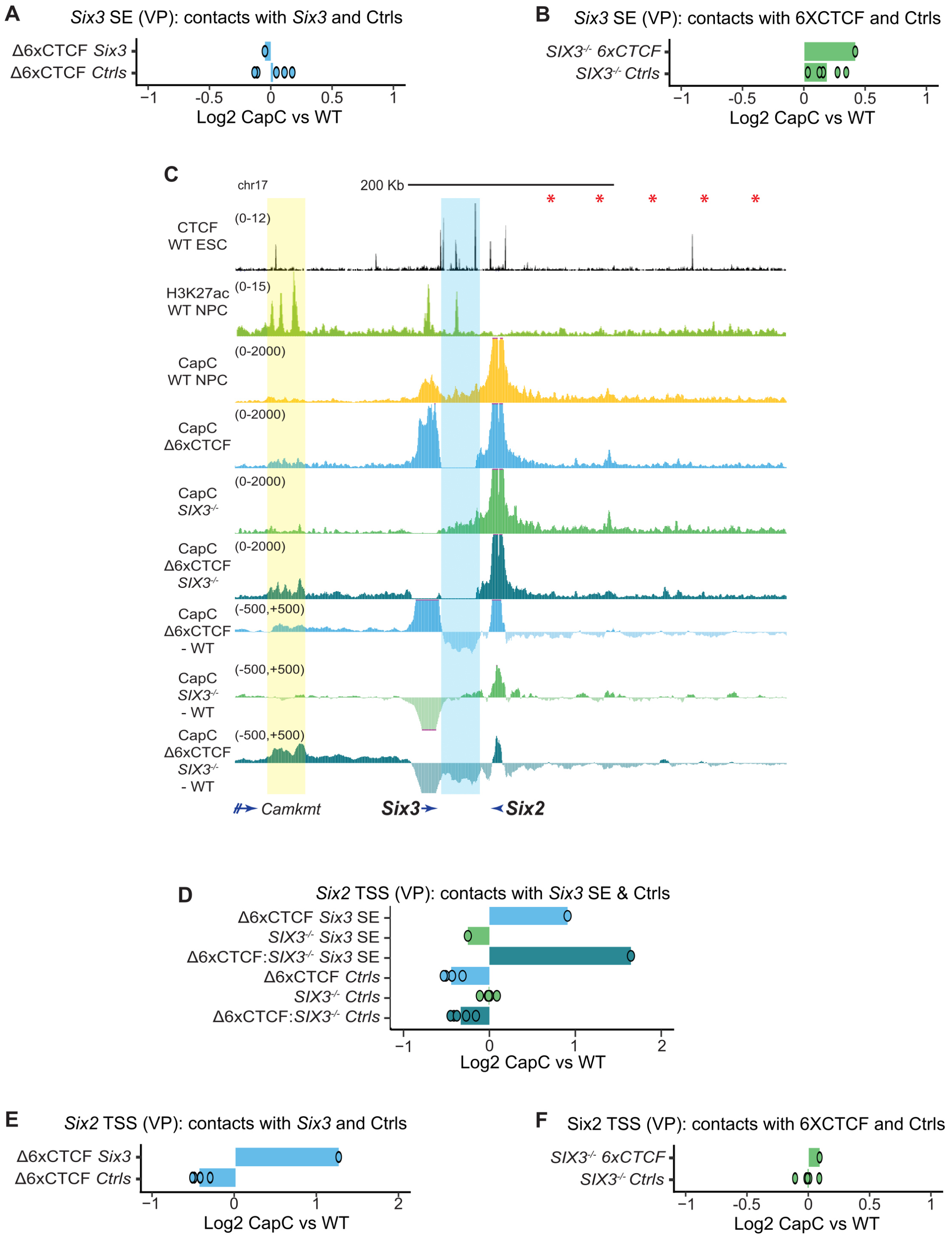

Supplementary Fig. 21: Capture-C experiments in Δ6XCTCF, Six3$^{-/-}$ and Δ6XCTCF:Six3$^{-/-}$ NPC. (A-B) *The average Capture-C signals shown in Fig. 6D (i.e. using the Six3 SE as a viewpoint) were measured within (A) the Six3 gene (chr17:85618333-85628691 (mm10)), (B) the 6XCTCF cluster (chr17:85629607-85666336 (mm10)) and within five different 30 Kb control regions (Ctrls) located within the Gbx2 TAD (red asterisks in Fig.6D indicate the midpoint of the following controls regions (mm10): chr17:85388601-85418601; chr17:85338601-85368601, chr17:85288601-85318601, chr17:85238601-85268601, chr17:85188601-85218601).* ***(C)*** *Capture-C experiments were performed as two biological replicates in WT, Δ6XCTCF, Six3$^{-/-}$ and Δ6XCTCF:Six3$^{-/-}$ NPC using the Six2 promoter as a*

*viewpoint. The average Capture-C signals of the two replicates performed for each cell line are shown around the Six3/Six2 locus either individually (upper tracks) or after subtracting the WT Capture-C signals (lower tracks), using in all cases the mm10 reference genome. The six CTCF sites deleted within the Six3/Six2 TAD boundary are highlighted in blue. (**D-F**) The average Capture-C signals shown in (C) were measured around (D) the Six3 SE (highlighted in yellow in (C); chr17:85453601-85491823 (mm10)), (E) the Six3 gene (chr17:85618333-85628691 (mm10)), (F) the 6XCTCF cluster (highlighted in blue in (C); (chr17:85629607-85666336 (mm10)) and within five different 30 Kb control regions (Ctrls) located within the Six2 TAD (red asterisks in (C) indicate the midpoint of the following controls regions (mm10): chr17:85723254-85753254, chr17:85773254-85803254, chr17:85823254-85853254, chr17:85873254-85903254, chr17:85923254-85953254). In (A-B) and (D-F), Capture-C signals are shown for the Δ6XCTCF, Six3$^{-/-}$ and Δ6XCTCF:Six3$^{-/-}$ NPC as log2 fold-changes with respect to WT NPC. In (A) and (E), the Capture-C signals were not measured around the Six3 gene in those cell lines in which the gene was deleted (i.e. Six3$^{-/-}$ and Δ6XCTCF:Six3$^{-/-}$). In (B) and (F), the Capture-C signals were not measured around the 6XCTCF cluster in those ESC lines in which that region was deleted (i.e. Δ6XCTCF and 6XCTCF:Six3$^{-/-}$).*

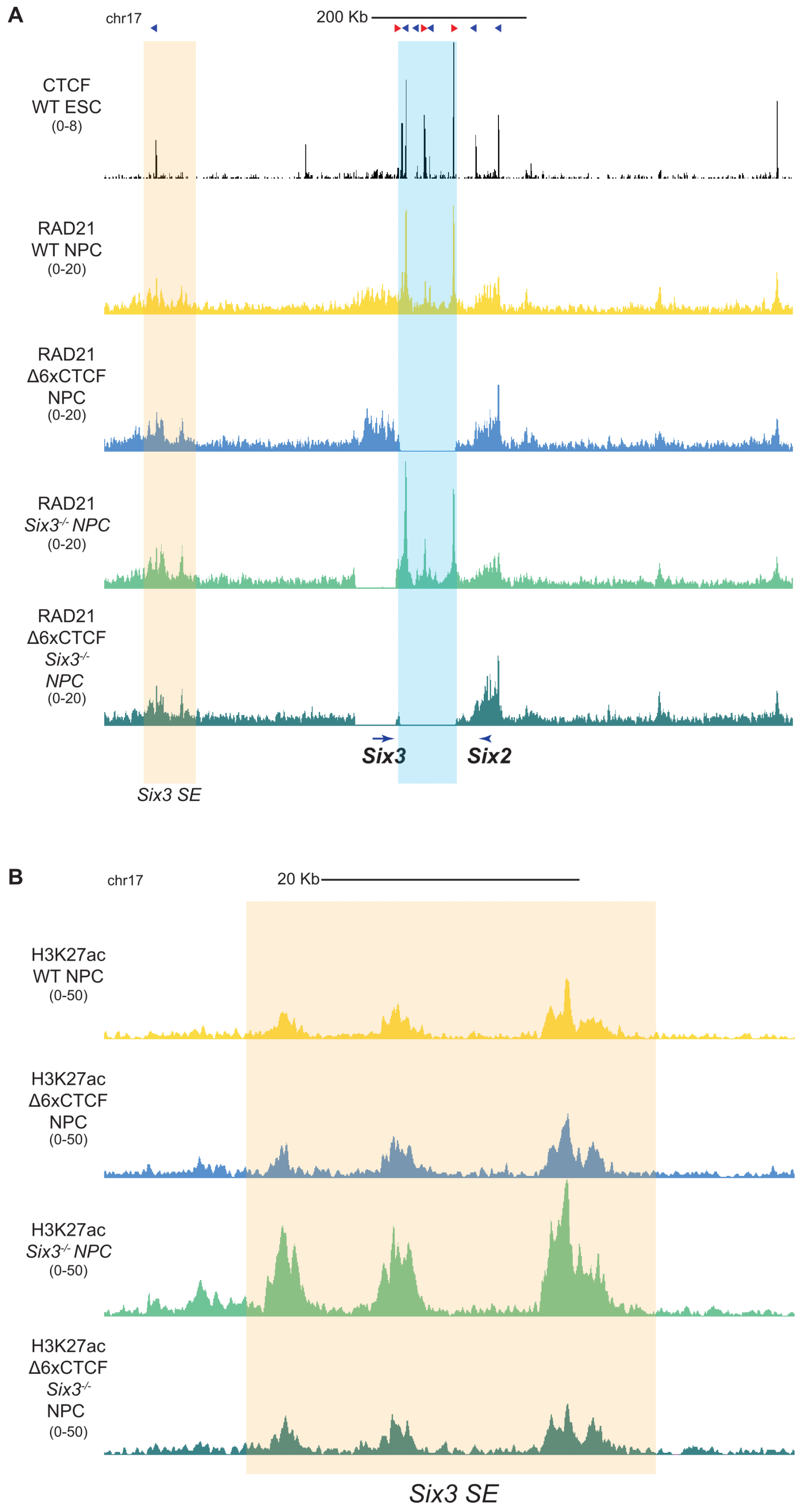

Supplementary Fig. 22: RAD21 and H3K27ac profiles in NPC with genomic re-arrangements within the Six3/Six2 locus. (A-B) *ChIP-seq profiles for RAD21 (A) and H3K27ac (B) are shown in NPC that are either WT or homozygous for the Δ6XCTCF, Six3$^{-/-}$ or Δ6XCTCF:Six3$^{-/-}$ deletions. In (A), CTCF ChiP-seq profiles generated in WT mESC [2] are also shown. The Six3 SE is highlighted in orange and the six CTCF sites deleted within the Six3/Six2 TAD boundary are highlighted in blue. The orientation of key CTCF sites is illustrated with red (sense) and blue (antisense) triangles.*

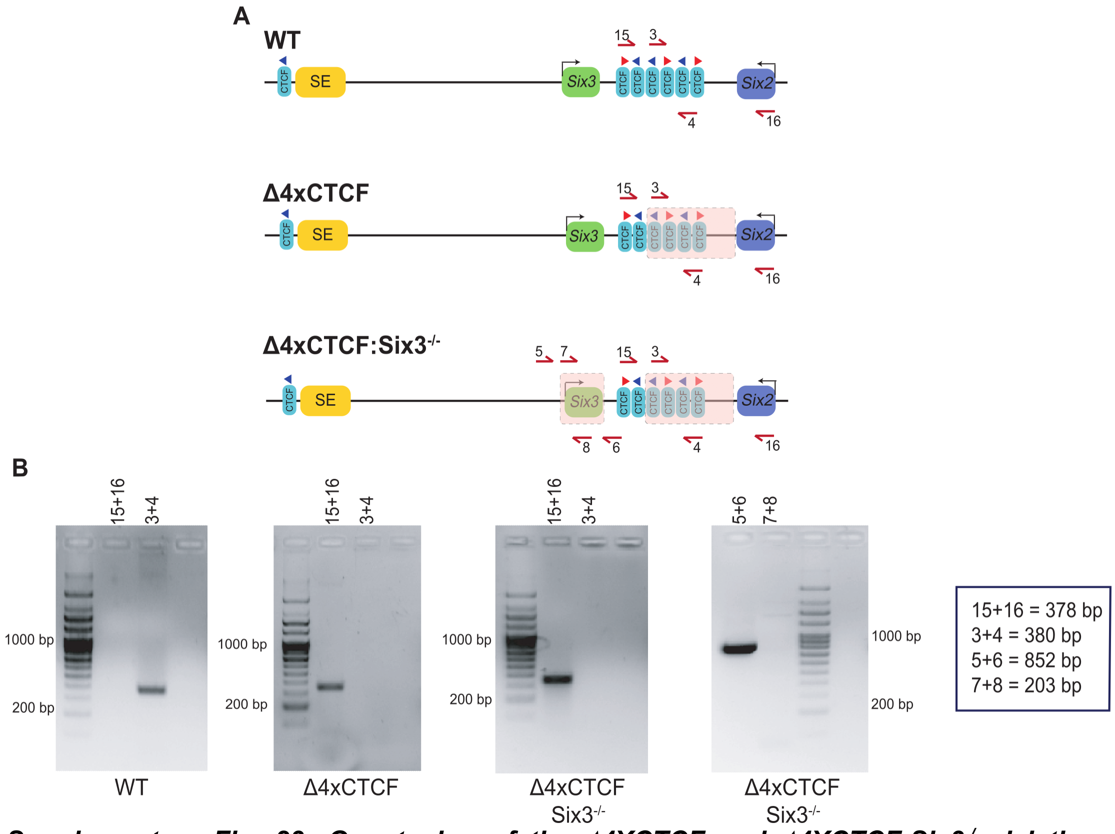

Supplementary Fig. 23: Genotyping of the Δ4XCTCF and Δ4XCTCF:Six3-/- deletions generated at the Six3/Six2 locus. (A) *Graphical overview of the PCR-based strategy used to genotype the Δ4XCTCF and Δ4XCTCF:Six3-/- deletions described in Fig 6. The horizontal arrows and accompanying numbers represent PCR primers.* ***(B)*** *Representative PCR genotyping results obtained for ESC lines that were either WT or homozygous for the Δ4XCTCF and Δ4XCTCF:Six3-/- deletions using the indicated primer pair combinations. The expected sizes of the amplicons obtained with each PCR primer combination are shown at the bottom right corner.*

Supplementary References

1. Bonev, B. *et al.* Multiscale 3D Genome Rewiring during Mouse Neural Development. *Cell* **171**, 557-572.e24 (2017).
2. Pope, B. D. *et al.* Topologically associating domains are stable units of replication-timing regulation. *Nature* **515**, 402–405 (2014).
3. Dixon, J. R. *et al.* Chromatin architecture reorganization during stem cell differentiation. *Nature* **518**, 331–336 (2015).
4. Lee, D. K. Alternatives to P value: confidence interval and effect size. *Korean J. Anesthesiol.* **69**, 555–562 (2016).
5. Hintermann, A. *et al.* Developmental and evolutionary comparative analysis of a regulatory landscape in mouse and chicken. *Development* **149**, dev200594 (2022).
6. Franke, M. *et al.* CTCF knockout in zebrafish induces alterations in regulatory landscapes and developmental gene expression. *Nat. Commun.* **12**, 5415 (2021).

Source Data (uncropped images for all gels shown in Supplementary Figures)

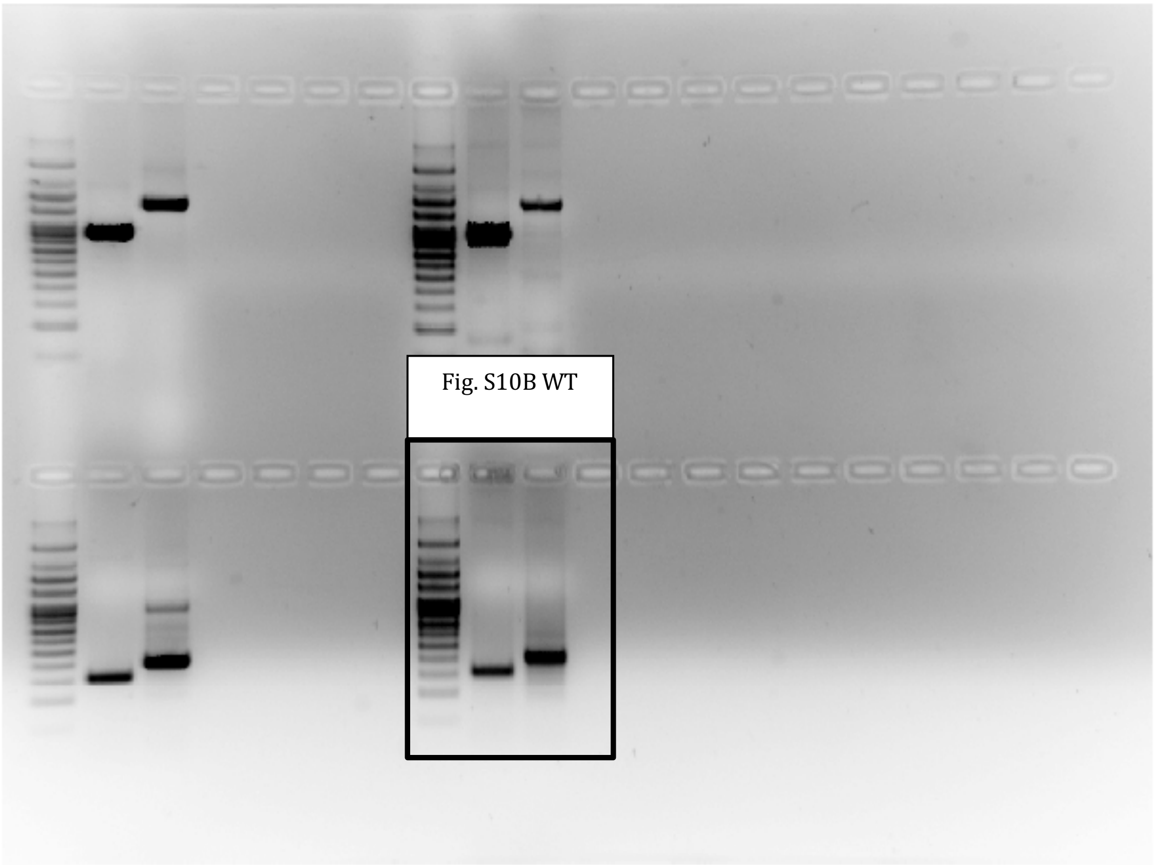

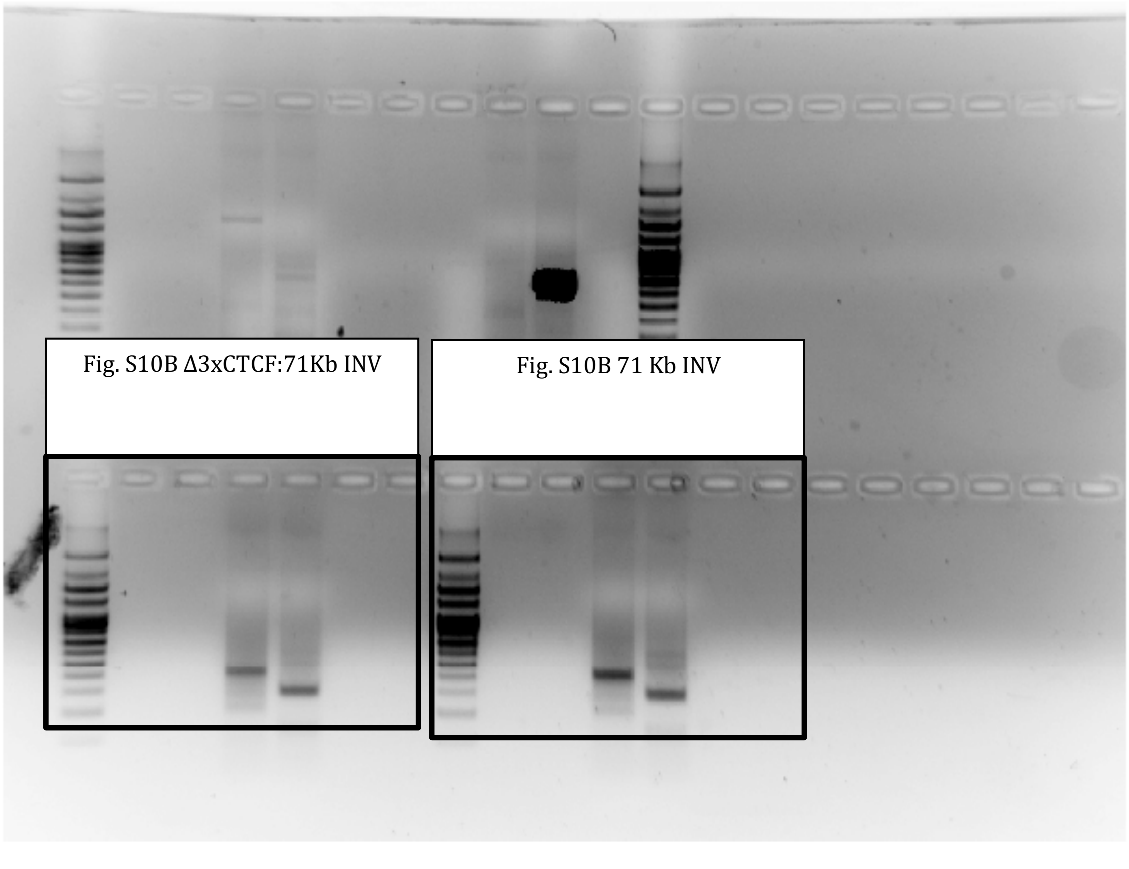

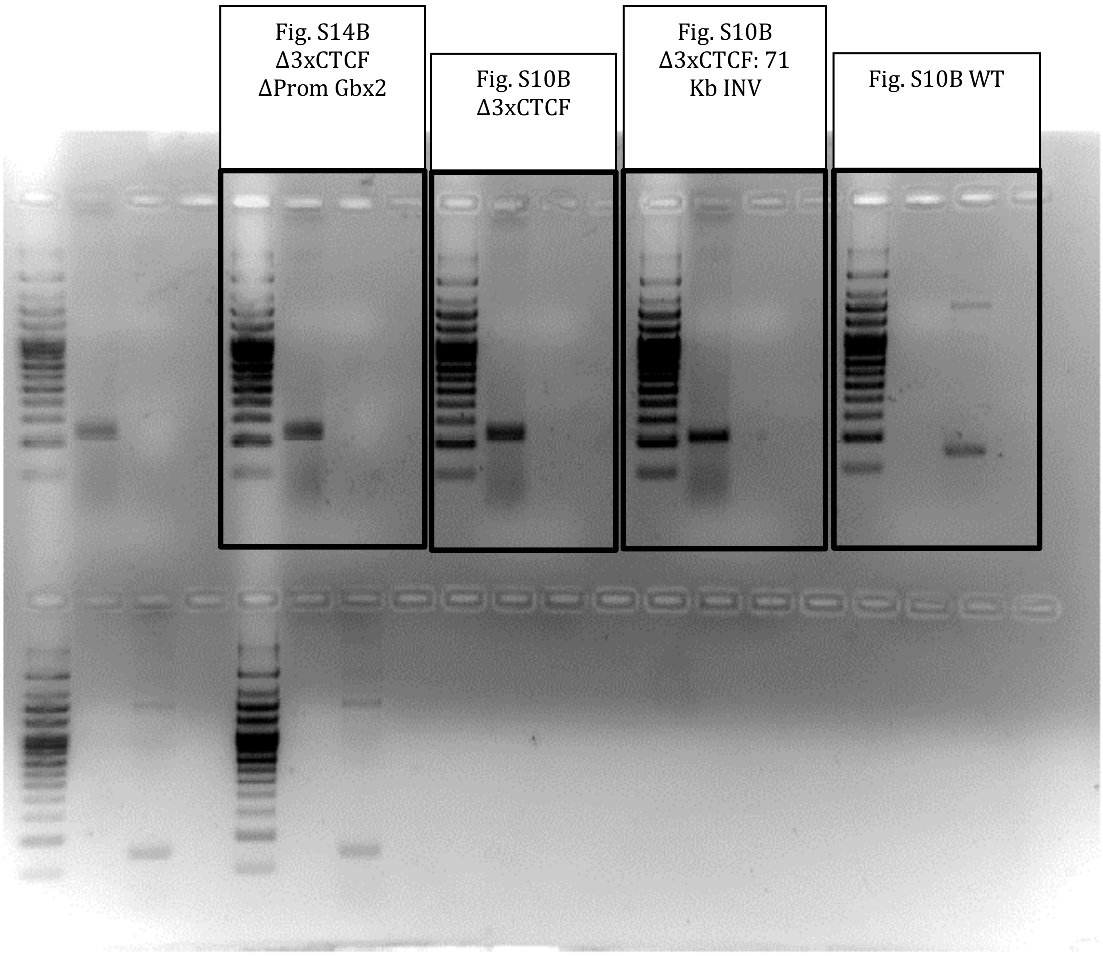
Fig. S14B
Δ3xCTCF
ΔProm Gbx2
Fig. S10B
Δ3xCTCF
Fig. S10B
Δ3xCTCF: 71
Kb INV
Fig. S10B WT

Fig. S11C ΔCTCF SE Gbx2 and WT

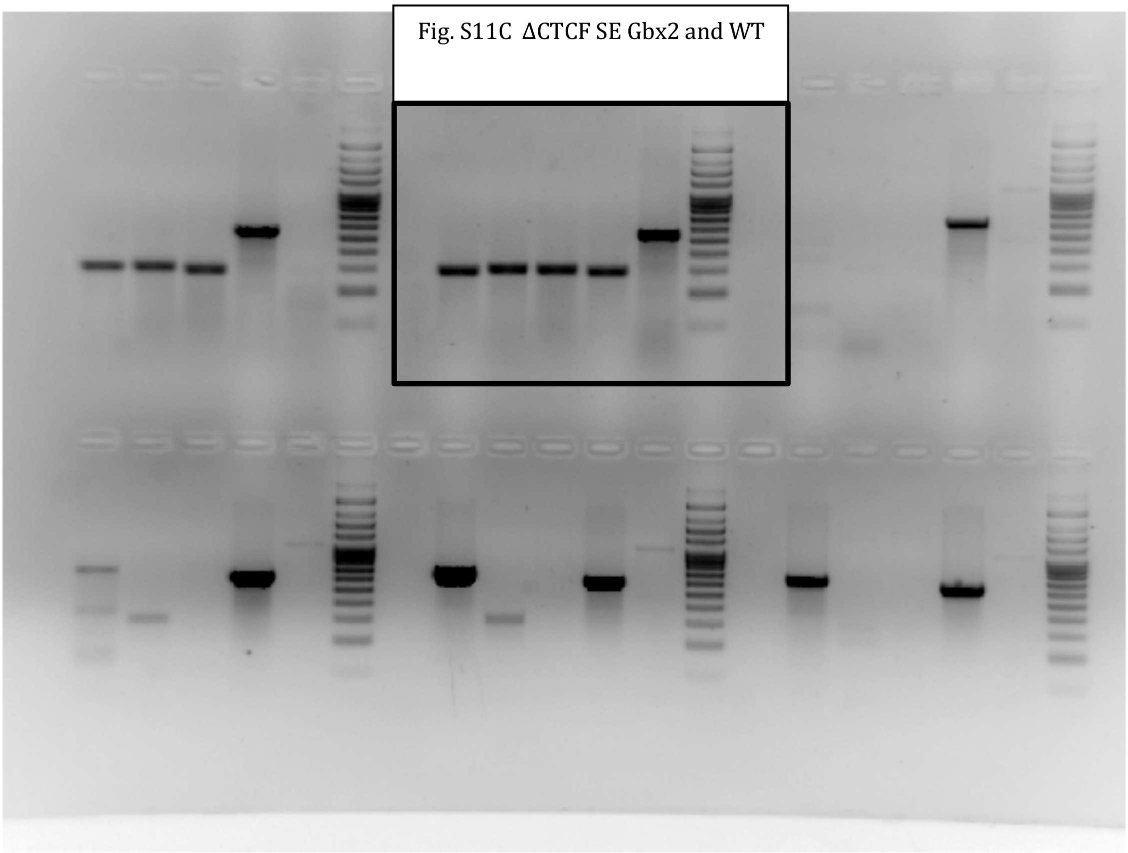

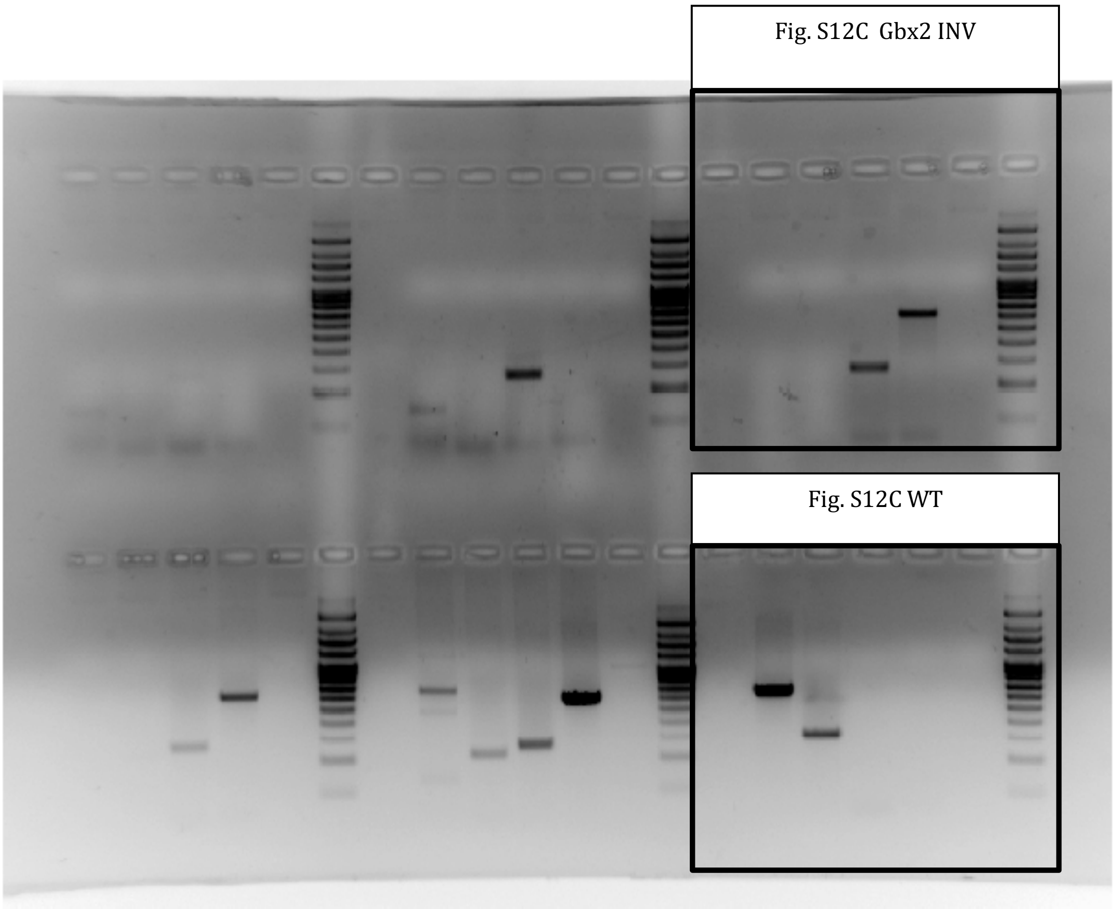
Fig. S12C Gbx2 INV
Fig. S12C WT

Fig. S12C Gbx2 INV:Δ3xCTCF

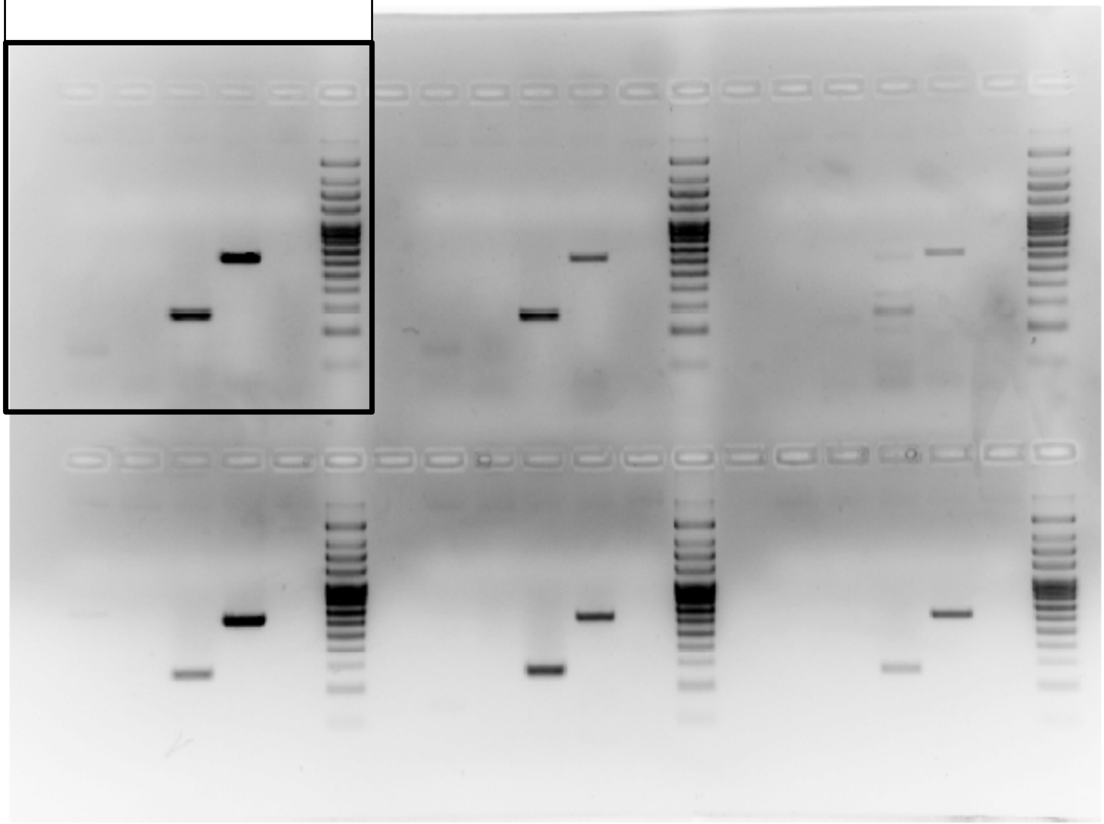

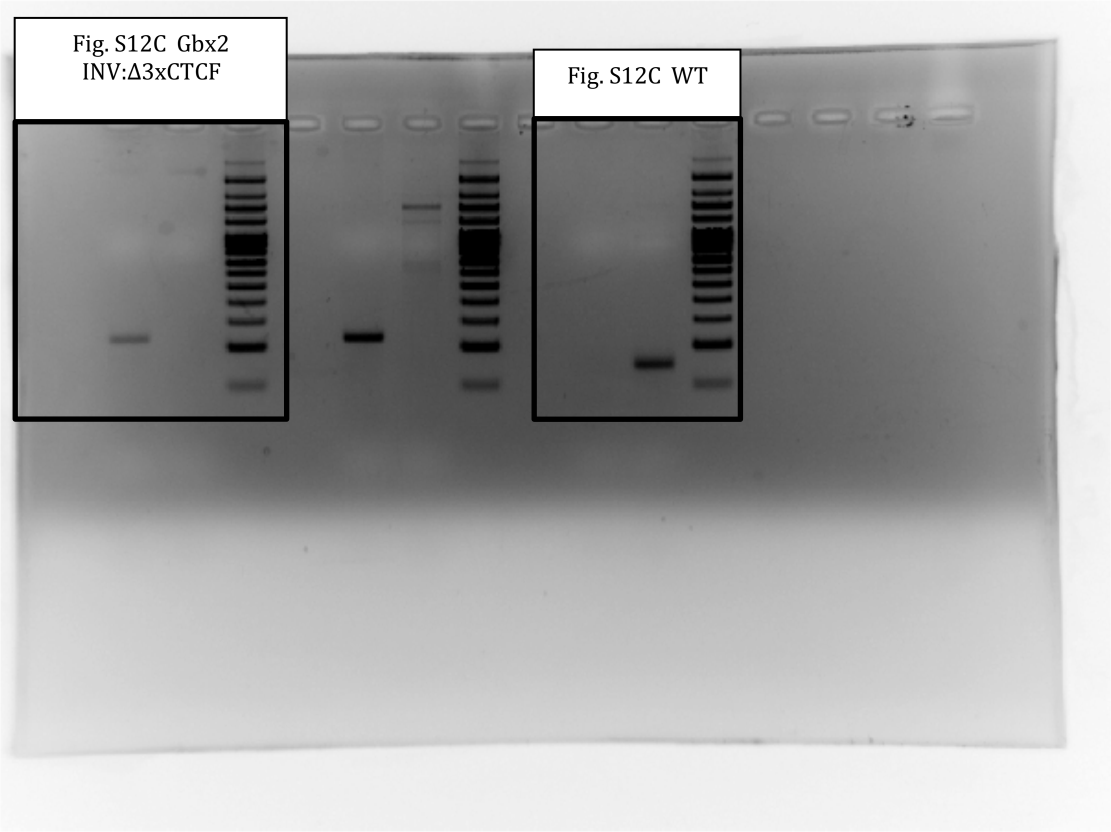
Fig. S12C Gbx2
INV:Δ3xCTCF
Fig. S12C WT

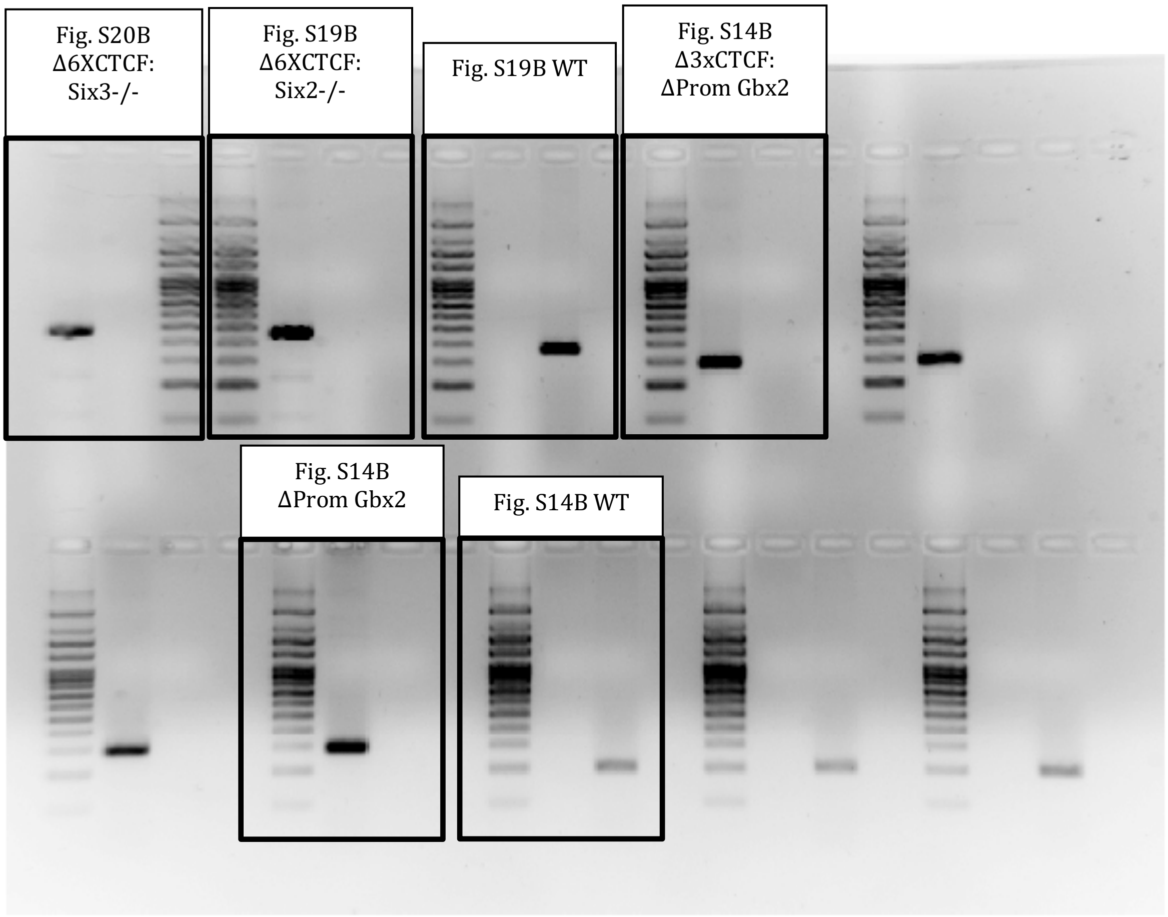
Fig. S20B
Δ6XCTCF:
Six3-/-
Fig. S19B
Δ6XCTCF:
Six2-/-
Fig. S19B WT
Fig. S14B
Δ3xCTCF:
ΔProm Gbx2
Fig. S14B
ΔProm Gbx2
Fig. S14B WT

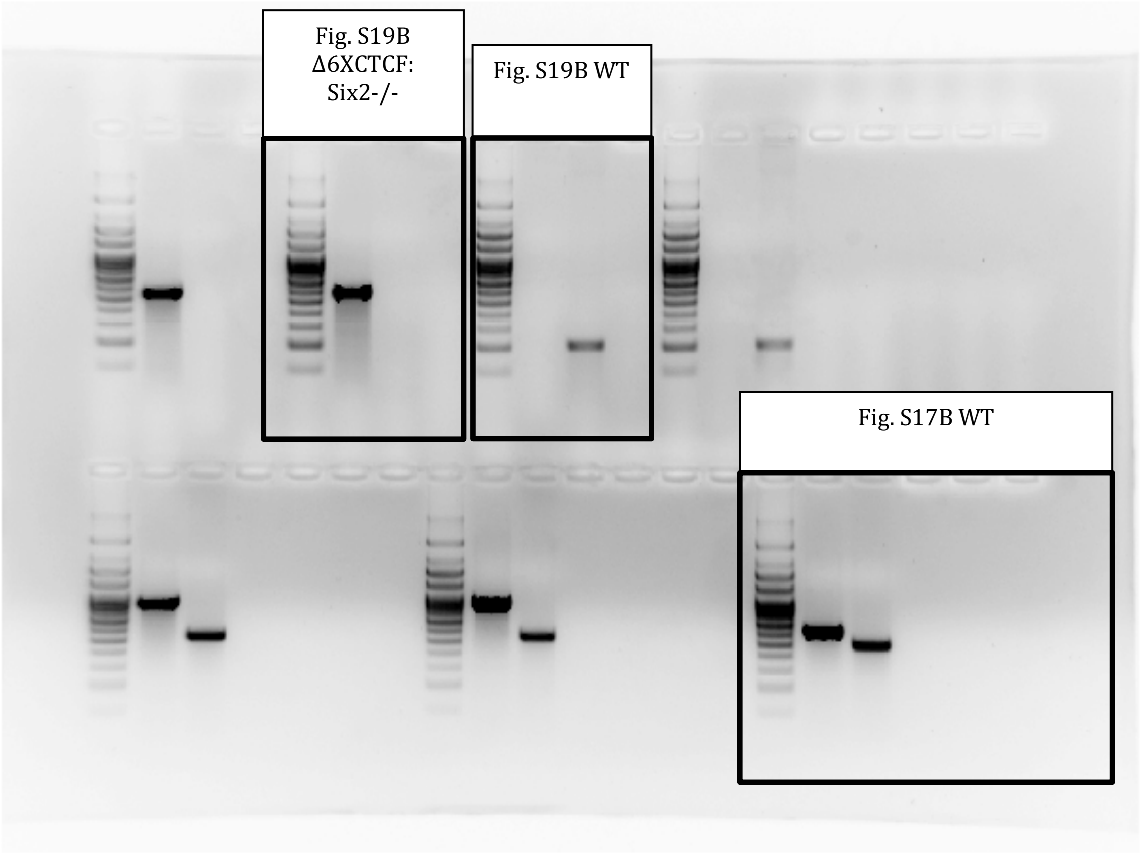
Fig. S19B Δ6XCTCF: Six2-/-
Fig. S19B WT
Fig. S17B WT

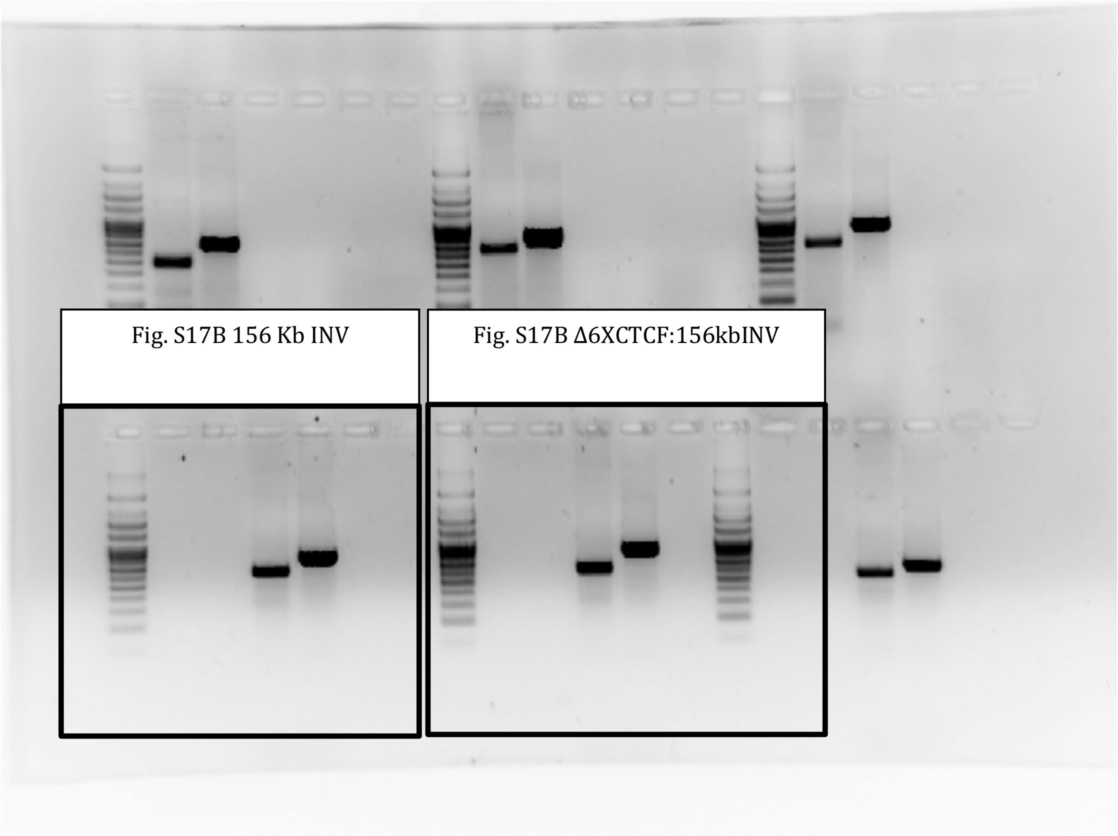
Fig. S17B 156 Kb INV
Fig. S17B Δ6XCTCF:156kbINV

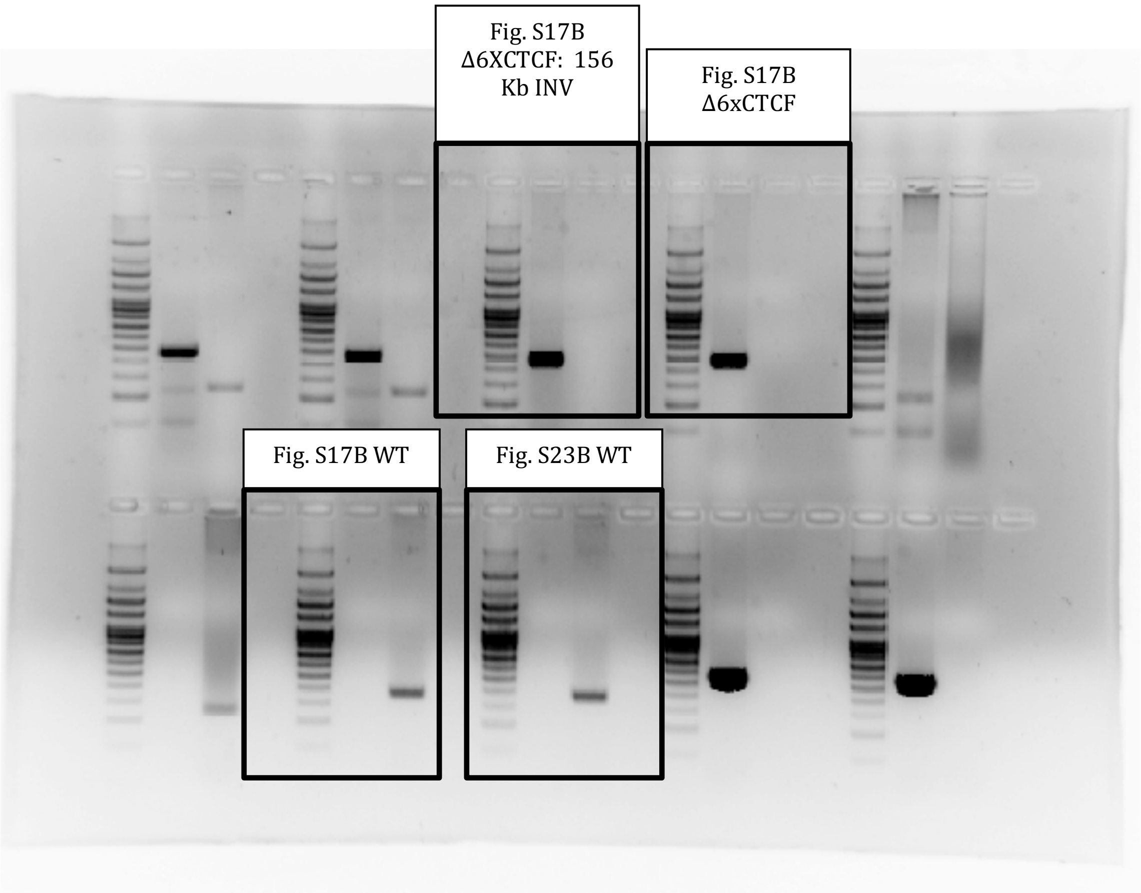
Fig. S17B Δ6XCTCF: 156 Kb INV
Fig. S17B Δ6xCTCF
Fig. S17B WT
Fig. S23B WT

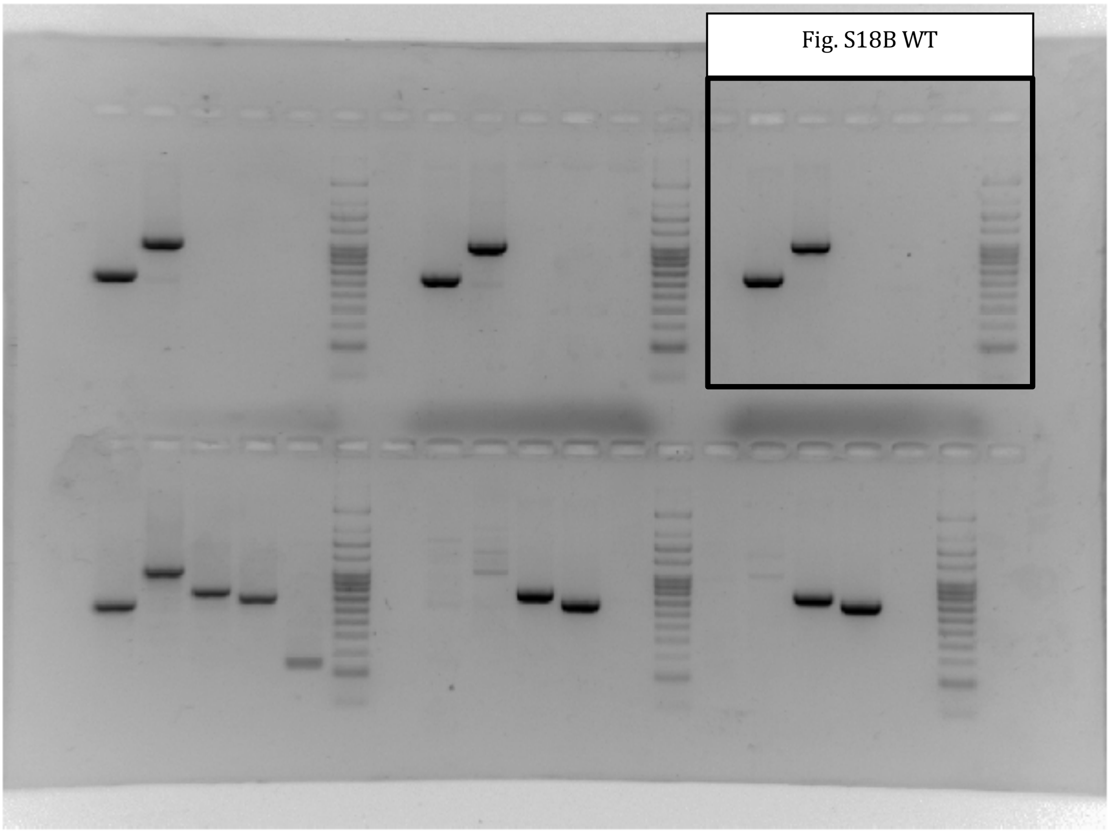
Fig. S18B WT

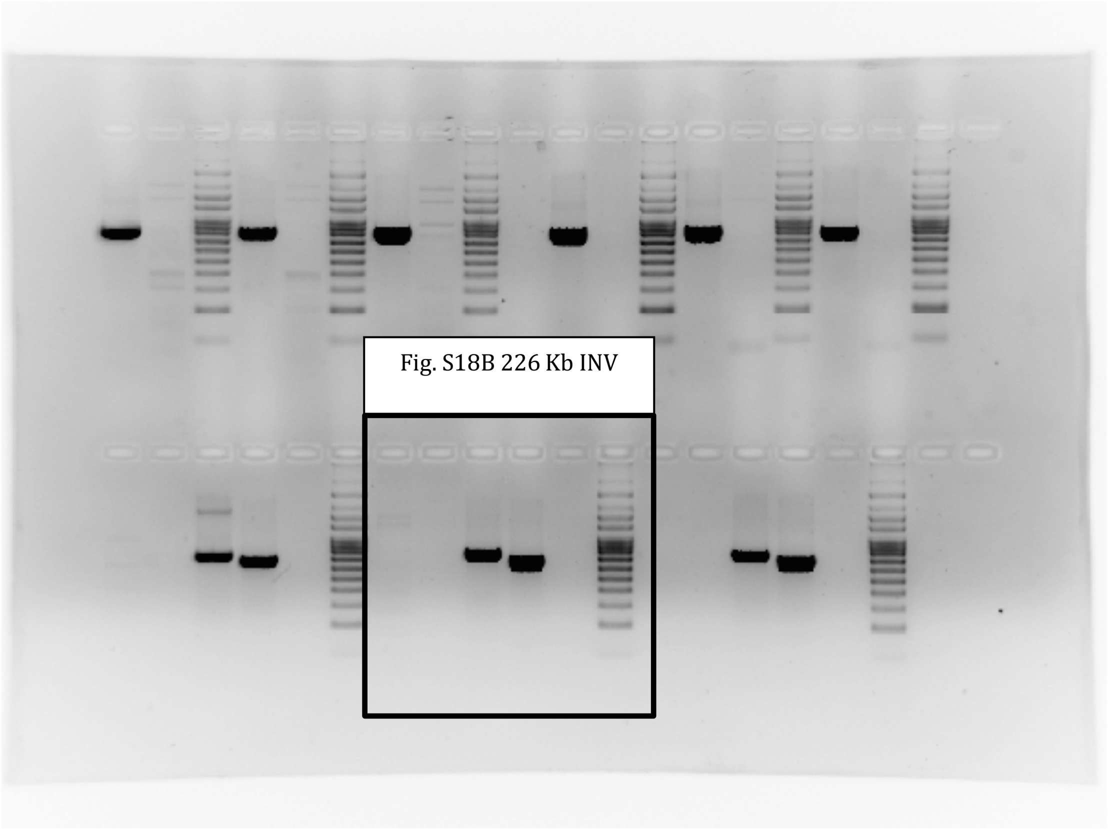
Fig. S18B 226 Kb INV

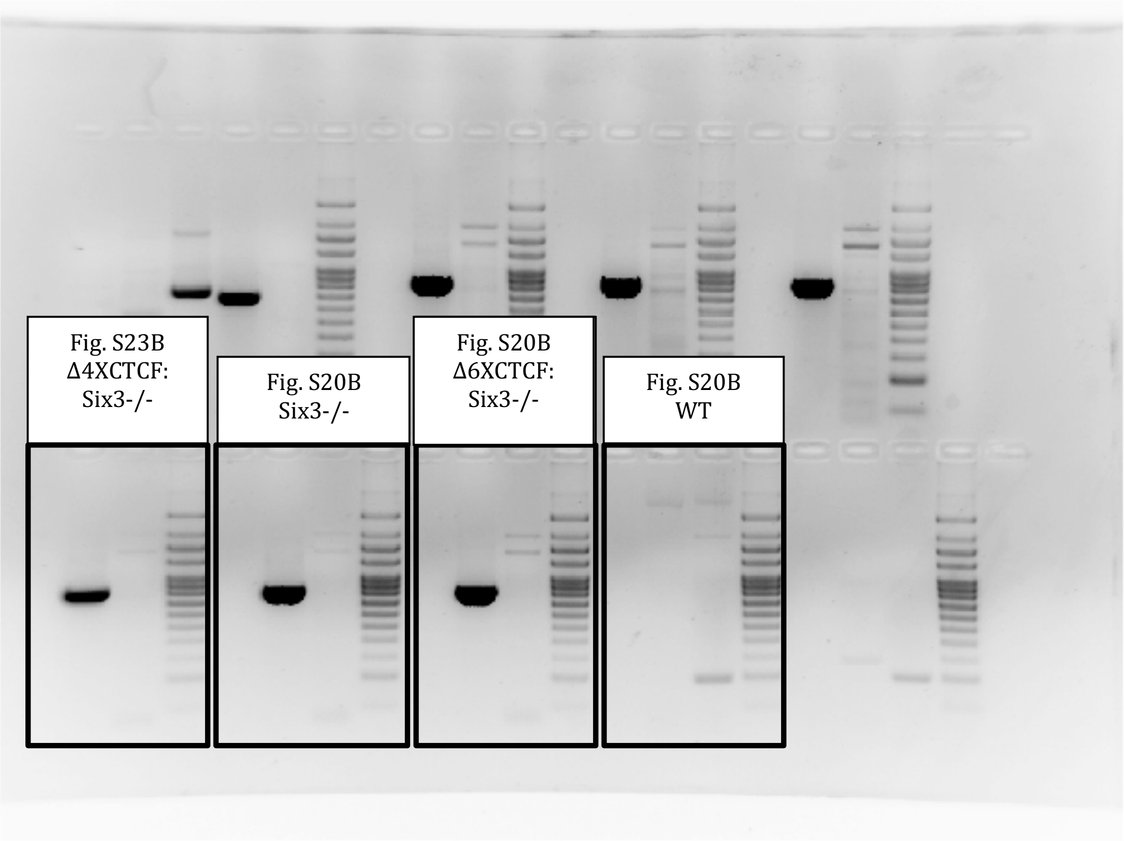
Fig. S23B
Δ4XCTCF:
Six3-/-
Fig. S20B
Six3-/-
Fig. S20B
Δ6XCTCF:
Six3-/-
Fig. S20B
WT

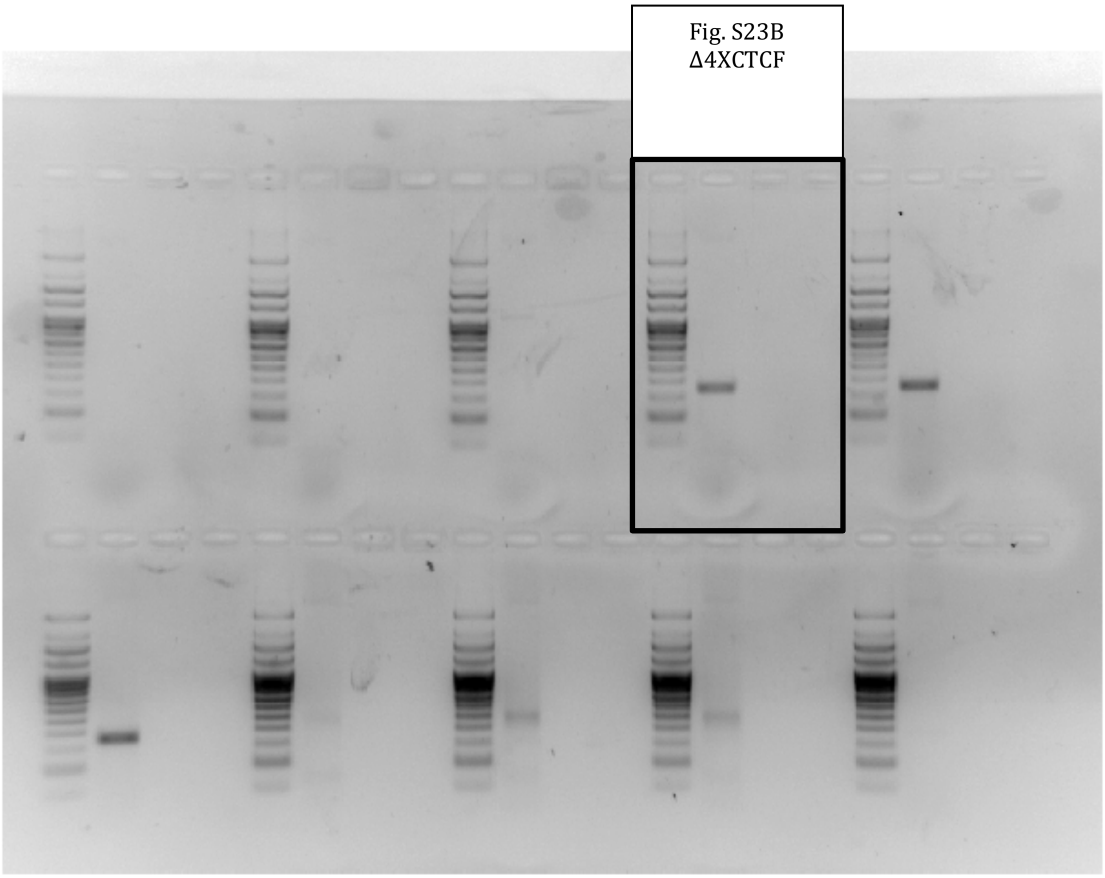
Fig. S23B
Δ4XCTCF

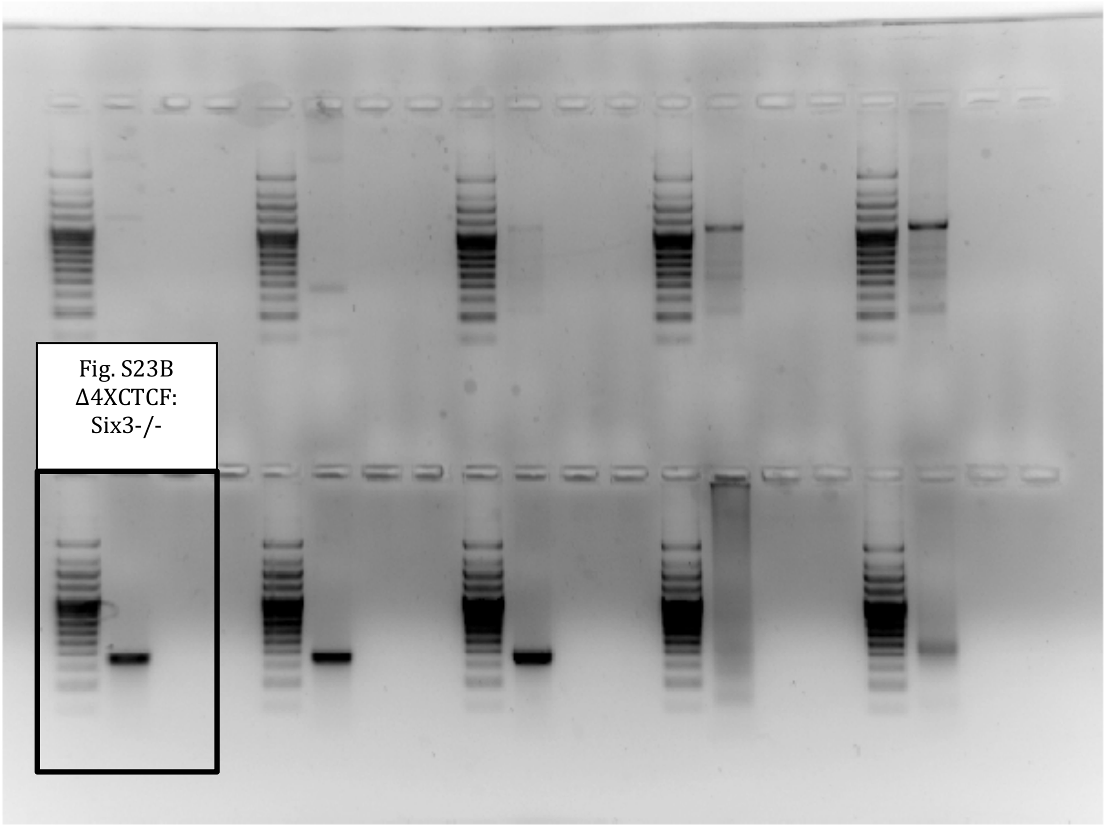
Fig. S23B
Δ4XCTCF:
Six3-/-
