# Supplementary material for: Cooperative insulation of regulatory domains by CTCF-dependent physical insulation and promoter competition
Source: Nat Commun. 2024 Aug 23;15:7258. doi: 10.1038/s41467-024-51602-4 (PMC11344162; doi:10.1038/s41467-024-51602-4)
Supplement: Supplementary file 3 — Description of Additional Supplementary Files [file 41467_2024_51602_MOESM3_ESM.pdf]

Description of Additional Supplementary Files

Supplementary Data 1. Gene ontology (GO) enrichment analysis for mouse genes located in TADs with different gene density.

Supplementary Data 2. Gene ontology (GO) enrichment analysis for human genes located in TADs with different gene density.

Supplementary Data 3. List of oligonucleotides (PCR primers, gRNAs and Capture-C probes) used in this study.
