# Supplementary material for: Cooperative insulation of regulatory domains by CTCF-dependent physical insulation and promoter competition
Source: Nat Commun. 2024 Aug 23;15:7258. doi: 10.1038/s41467-024-51602-4 (PMC11344162; doi:10.1038/s41467-024-51602-4)
Supplement: Supplementary file 7 — Reporting Summary [file 41467_2024_51602_MOESM7_ESM.pdf]

nature portfolio

Corresponding author(s): Victor Sanchez-Gaya
Endika Haro
Alvaro Rada-Iglesias

Last updated by author(s): Jul 31, 2024

Reporting Summary

Nature Portfolio wishes to improve the reproducibility of the work that we publish. This form provides structure for consistency and transparency in reporting. For further information on Nature Portfolio policies, see our [Editorial Policies](#) and the [Editorial Policy Checklist](#).

Statistics

For all statistical analyses, confirm that the following items are present in the figure legend, table legend, main text, or Methods section.

n/a	Confirmed	
☐	☒	The exact sample size (n) for each experimental group/condition, given as a discrete number and unit of measurement
☐	☒	A statement on whether measurements were taken from distinct samples or whether the same sample was measured repeatedly
☐	☒	The statistical test(s) used AND whether they are one- or two-sided<br>*Only common tests should be described solely by name; describe more complex techniques in the Methods section.*
☒	☐	A description of all covariates tested
☐	☒	A description of any assumptions or corrections, such as tests of normality and adjustment for multiple comparisons
☐	☒	A full description of the statistical parameters including central tendency (e.g. means) or other basic estimates (e.g. regression coefficient) AND variation (e.g. standard deviation) or associated estimates of uncertainty (e.g. confidence intervals)
☐	☒	For null hypothesis testing, the test statistic (e.g. F, t, r) with confidence intervals, effect sizes, degrees of freedom and P value noted<br>*Give P values as exact values whenever suitable.*
☒	☐	For Bayesian analysis, information on the choice of priors and Markov chain Monte Carlo settings
☒	☐	For hierarchical and complex designs, identification of the appropriate level for tests and full reporting of outcomes
☐	☒	Estimates of effect sizes (e.g. Cohen's d, Pearson's r), indicating how they were calculated

Our web collection on [statistics for biologists](#) contains articles on many of the points above.

Software and code

Policy information about [availability of computer code](#)

Data collection	RT-qPCR: CFX 384 detection system (Bio-Rad)<br>Next-generation sequencing: NovaSeq 6000 System (Illumina)
Data analysis	fastqc 0.11.8<br>MultiQC 1.0.dev0<br>trimmomatic 0.38<br>Bowtie2 2.2.5<br>SAMtools 1.9<br>deepTools 3.1.3<br>capC-MAP 1.1.3<br>bedGraphToBigWig 2.10<br>bigWigAverageOverBed 2<br>bigWigMerge 2<br>bedtools 2.27.1<br>Housekeeping and Reference Transcript Atlas database 1.0<br>MACS2 2.1.1.20160309<br>cooltools 0.4.0-dev<br>hic2cool 0.8.3<br>R 4.3.2<br>WebGestalt R package 0.4.6<br>Inflection R package 1.3.6

CTCF R package 0.99.11
UCSC genome browser (https://genome.ucsc.edu/)
AMIGO browser (https://amigo.geneontology.org/amigo/)
3D Genome browser (http://3dgenome.fsm.northwestern.edu/)
4DN Data portal (https://data.4dnucleome.org/)
rcompanion R package 2.4.36

For manuscripts utilizing custom algorithms or software that are central to the research but not yet described in published literature, software must be made available to editors and reviewers. We strongly encourage code deposition in a community repository (e.g. GitHub). See the Nature Portfolio guidelines for submitting code & software for further information.

Data

Policy information about availability of data

All manuscripts must include a data availability statement. This statement should provide the following information, where applicable:

- Accession codes, unique identifiers, or web links for publicly available datasets
- A description of any restrictions on data availability
- For clinical datasets or third party data, please ensure that the statement adheres to our policy

All the generated ChIP-seq data are publically available through GEO (GSE252218).
All the generated Capture-C data are publically available through GEO (GSE252080).

Research involving human participants, their data, or biological material

Policy information about studies with human participants or human data. See also policy information about sex, gender (identity/presentation), and sexual orientation and race, ethnicity and racism.

Reporting on sex and gender	NA
Reporting on race, ethnicity, or other socially relevant groupings	NA
Population characteristics	NA
Recruitment	NA
Ethics oversight	NA

Note that full information on the approval of the study protocol must also be provided in the manuscript.

Field-specific reporting

Please select the one below that is the best fit for your research. If you are not sure, read the appropriate sections before making your selection.

☒ Life sciences ☐ Behavioural & social sciences ☐ Ecological, evolutionary & environmental sciences

For a reference copy of the document with all sections, see nature.com/documents/nr-reporting-summary-flat.pdf

Life sciences study design

All studies must disclose on these points even when the disclosure is negative.

Sample size	For all the transgenic ESC lines we generated at least two clonal lines with the same genotype. Taking into account the amount of different cell lines generated and that the clones from the same cell line were generally responding in a similar manner, we decided to keep a minimum of two clones per investigated genotype.
Data exclusions	No data was excluded.
Replication	All experiments were performed independently at least twice for each clonal line (i.e. at least four biological replicates per genotype) and all the attempts at data replication were successful. The exact number of biological replicates is described in the corresponding figure legends.
Randomization	Randomization was not relevant for our study as the sample sizes of the different experiments were too small for randomization.
Blinding	The investigators were not blinded to allocation during experiments and outcome assessment. There was one person in charge of generating and characterizing all the transgenic cell lines and we did not have additional personal that could be solely in charge of analyzing the data. Blinding is typically used with randomization and large sample sizes, which, as stated before, does not apply to our experiments.

Behavioural & social sciences study design

All studies must disclose on these points even when the disclosure is negative.

Study description	NA
Research sample	NA
Sampling strategy	NA
Data collection	NA
Timing	NA
Data exclusions	NA
Non-participation	NA
Randomization	NA

Ecological, evolutionary & environmental sciences study design

All studies must disclose on these points even when the disclosure is negative.

Study description	NA
Research sample	NA
Sampling strategy	NA
Data collection	NA
Timing and spatial scale	NA
Data exclusions	NA
Reproducibility	NA
Randomization	NA
Blinding	NA

Did the study involve field work? ☐ Yes ☒ No

Field work, collection and transport

Field conditions	NA
Location	NA
Access & import/export	NA
Disturbance	NA

Reporting for specific materials, systems and methods

We require information from authors about some types of materials, experimental systems and methods used in many studies. Here, indicate whether each material, system or method listed is relevant to your study. If you are not sure if a list item applies to your research, read the appropriate section before selecting a response.

Materials & experimental systems

n/a	Involved in the study
☐	☒ Antibodies
☐	☒ Eukaryotic cell lines
☒	☐ Palaeontology and archaeology
☒	☐ Animals and other organisms
☒	☐ Clinical data
☒	☐ Dual use research of concern
☒	☐ Plants

Methods

n/a	Involved in the study
☐	☒ ChIP-seq
☒	☐ Flow cytometry
☒	☐ MRI-based neuroimaging

Antibodies

Antibodies used

RAD21 (abcam, ab154769, lot#GR32241) ; 10 µg /ChIP-seq sample
H3K27ac (Active Motif, 39133, lot#31521015); 3 µg /ChIP-seq sample

Validation

RAD21 antibody was validate by the manufacturer: Rhodes JDP et al., Cohesin Disrupts Polycomb-Dependent Chromosome Interactions in Embryonic Stem Cells. Cell Rep. 2020 Jan 21;30(3):820-835.e10. doi: 10.1016/j.celrep.2019.12.057. PMID: 31968256; PMCID: PMC6988126.
H3K27ac antibody was validated in: Cruz-Molina, S. et al. PRC2 Facilitates the Regulatory Topology Required for Poised Enhancer Function during Pluripotent Stem Cell Differentiation. Cell Stem Cell 20, 1–17 (2017).

Eukaryotic cell lines

Policy information about cell lines and Sex and Gender in Research

Cell line source(s)

Male mouse ESC line E14Tg2a was used for all experiments. This cell line was a kind gift from Joanna Wysocka´s lab (Standford University). All the rearrangements were done using CRISPR/Cas9 and the gRNAs are described in Supplementary Data 3. Using the E14Tg2a mESC, the following cell lines were generated in this work:

Gbx2/Asb18 locus: Δ3xCTCF, 71 Kb INV, Δ3xCTCF:71 Kb INV, ΔProm Gbx2, Δ3xCTCF:ΔProm Gbx2, ΔCTCF SE Gbx2, Gbx2 INV, Gbx2 INV:Δ3xCTCF

Six3/Six2 locus: Δ6xCTCF, 156 Kb INV, Δ6xCTCF:156 Kb INV, Six3-/-, Δ6xCTCF:Six3-/-, Δ4xCTCF, Δ4xCTCF:Six3-/-, Δ6xCTCF:Six2-/-, 226 Kb INV

Authentication

All the rearrangements were authenticated by PCR genotyping (see supplementary figures)

Mycoplasma contamination

The WT E14 mESC are regularly tested and no contamination has been detected.

Commonly misidentified lines
(See ICLAC register)

None

Palaeontology and Archaeology

Specimen provenance

NA

Specimen deposition

NA

Dating methods

NA

☐ Tick this box to confirm that the raw and calibrated dates are available in the paper or in Supplementary Information.

Ethics oversight

NA

Note that full information on the approval of the study protocol must also be provided in the manuscript.

Animals and other research organisms

Policy information about studies involving animals; ARRIVE guidelines recommended for reporting animal research, and Sex and Gender in Research

Laboratory animals

NA

Wild animals

NA

Reporting on sex	NA
Field-collected samples	NA
Ethics oversight	NA

Note that full information on the approval of the study protocol must also be provided in the manuscript.

Clinical data

Policy information about clinical studies

All manuscripts should comply with the ICMJE guidelines for publication of clinical research and a completed CONSORT checklist must be included with all submissions.

Clinical trial registration	NA
Study protocol	NA
Data collection	NA
Outcomes	NA

Dual use research of concern

Policy information about dual use research of concern

Hazards

Could the accidental, deliberate or reckless misuse of agents or technologies generated in the work, or the application of information presented in the manuscript, pose a threat to:

No	Yes	
☒	☐	Public health
☒	☐	National security
☒	☐	Crops and/or livestock
☒	☐	Ecosystems
☒	☐	Any other significant area

Experiments of concern

Does the work involve any of these experiments of concern:

No	Yes	
☒	☐	Demonstrate how to render a vaccine ineffective
☒	☐	Confer resistance to therapeutically useful antibiotics or antiviral agents
☒	☐	Enhance the virulence of a pathogen or render a nonpathogen virulent
☒	☐	Increase transmissibility of a pathogen
☒	☐	Alter the host range of a pathogen
☒	☐	Enable evasion of diagnostic/detection modalities
☒	☐	Enable the weaponization of a biological agent or toxin
☒	☐	Any other potentially harmful combination of experiments and agents

Plants

Seed stocks

NA

Novel plant genotypes

NA

Authentication

NA

ChIP-seq

Data deposition

☒ Confirm that both raw and final processed data have been deposited in a public database such as GEO.

☐ Confirm that you have deposited or provided access to graph files (e.g. BED files) for the called peaks.

Data access links
May remain private before publication.

https://www.ncbi.nlm.nih.gov/geo/query/acc.cgi?acc=GSE252218

Files in database submission

GSM7996810 WT_ESC_RAD21
GSM7996811 WT_ESC_H3K27ac
GSM7996812 Δ3XCTCF_ESC_RAD21
GSM7996813 Δ3XCTCF_ESC_H3K27ac
GSM7996814 ΔPromGbx2_ESC_RAD21
GSM7996815 ΔPromGbx2_ESC_H3K27ac
GSM7996816 Δ3XCTCF_ΔPromGbx2_ESC_RAD21
GSM7996817 Δ3XCTCF_ΔPromGbx2_ESC_H3K27ac
GSM7996818 WT_NPC_RAD21
GSM7996819 WT_NPC_H3K27ac
GSM7996820 Δ6XCTCF_NPC_RAD21
GSM7996821 Δ6XCTCF_NPC_H3K27ac
GSM7996822 Six3KO_NPC_RAD21
GSM7996823 Six3KO_NPC_H3K27ac
GSM7996824 Δ6XCTCF_Six3KO_NPC_RAD21
GSM7996825 Δ6XCTCF_Six3KO_NPC_H3K27ac

Genome browser session
(e.g. UCSC)

BigWig files are provided through GEO (GSE252218) for all generated ChIP-seq samples. BigWig files can be easily uploaded in the UCSC browser for visualization purposes.

Methodology

Replicates

Chip-seq experiments were performed as single replicates for each condition.

Sequencing depth

Truseq ChIP-seq + Novaseq6000_150PE (2x150bp) _40Mrds/spl

Antibodies

RAD21 (abcam, ab154769, lot#GR32241) ; 10 µg /ChIP-seq sample
H3K27ac (Active Motif, 39133, lot#31521015); 3 µg /ChIP-seq sample

Peak calling parameters

Peak calling was not performed

Data quality

Basic read quality check was performed using FastQC (Babraham Bioinformatics) and MultiQC. The removal of read adapters and low-quality filtering was done with trimmomatic.

Software

Reads were mapped to the mm10 reference genome with Bowtie2 and duplicated reads were discarded with SAMtools. Bigwig files were generated with bamCoverage from deepTools applying the reads per genome coverage normalization

Flow Cytometry

Plots

Confirm that:

- ☐ The axis labels state the marker and fluorochrome used (e.g. CD4-FITC).
- ☐ The axis scales are clearly visible. Include numbers along axes only for bottom left plot of group (a 'group' is an analysis of identical markers).
- ☐ All plots are contour plots with outliers or pseudocolor plots.
- ☐ A numerical value for number of cells or percentage (with statistics) is provided.

Methodology

Sample preparation	NA
Instrument	NA
Software	NA
Cell population abundance	NA
Gating strategy	NA

☐ Tick this box to confirm that a figure exemplifying the gating strategy is provided in the Supplementary Information.

Magnetic resonance imaging

Experimental design

Design type	NA
Design specifications	NA
Behavioral performance measures	NA

Acquisition

Imaging type(s)	NA
Field strength	NA
Sequence & imaging parameters	NA
Area of acquisition	NA

Diffusion MRI ☐ Used ☒ Not used

Preprocessing

Preprocessing software	NA
Normalization	NA
Normalization template	NA
Noise and artifact removal	NA
Volume censoring	NA

Statistical modeling & inference

Model type and settings	NA
Effect(s) tested	NA

Specify type of analysis: ☐ Whole brain ☐ ROI-based ☐ Both

Statistic type for inference

(See Eklund et al. 2016)

NA

Correction

NA

Models & analysis

n/a	Involved in the study
☒	☐ Functional and/or effective connectivity
☒	☐ Graph analysis
☒	☐ Multivariate modeling or predictive analysis

Functional and/or effective connectivity

NA

Graph analysis

NA

Multivariate modeling and predictive analysis

NA
